# Adaptive Output Containment Tracking Control for Heterogeneous Wide-Area Networks with Aperiodic Intermittent Communication and Uncertain Leaders

**DOI:** 10.3390/s23208631

**Published:** 2023-10-22

**Authors:** Yanpeng Shi, Jiangping Hu, Bijoy Kumar Ghosh

**Affiliations:** 1 School of Automation Engineering, University of Electronic Science and Technology of China, Chengdu 611731, China; ypshi@std.uestc.edu.cn (Y.S.); bijoy.ghosh@ttu.edu (B.K.G.); 2 Yangtze Delta Region Institute (Huzhou), University of Electronic Science and Technology of China, Huzhou 313001, China; 3 Department of Mathematics and Statistics, Texas Tech University, Lubbock, TX 79409-1042, USA

**Keywords:** heterogeneous clustered network, output containment, aperiodic intermittent control, adaptive estimation, average dwell-time condition

## Abstract

This paper proposes an adaptive distributed hybrid control approach to investigate the output containment tracking problem of heterogeneous wide-area networks with intermittent communication. First, a clustered network is modeled for a wide-area scenario. An aperiodic intermittent communication mechanism is exerted on the clusters such that clusters only communicate through leaders. Second, in order to remove the assumption that each follower must know the system matrix of the leaders and achieve output containment, a distributed adaptive hybrid control strategy is proposed for each agent under the internal model and adaptive estimation mechanism. Third, sufficient conditions based on average dwell-time are provided for the output containment achievement using a Lyapunov function method, from which the exponential stability of the closed-loop system is analyzed. Finally, simulation results are presented to demonstrate the effectiveness of the proposed adaptive distributed intermittent control strategy.

## 1. Introduction

Multi-agent systems in distributed cooperative settings have been a research focus because of their widespread applications, including spacecraft formation flying [1], mobile robots [2], and sensor networks [3]. An increasing number of studies consider various cooperative control problems under two types of network frameworks: leaderless and leader-following [4,5]. In the leader-following framework involving consensus with only one leader, a set of agents must reach the tracking trajectory of interest. In some real-world scenarios, agents are not forced to reach the same value or trajectory. As a special class of cooperative controls, containment control aims to drive all followers into a desirable region formed by multiple independent leaders [6]. In general, multi-agent systems can be divided into two broad classes according to their dynamics: homogeneous and heterogeneous [7,8]. Homogeneity signifies that the dynamics of the agents are identical, whereas heterogeneity signifies nonidentical dynamics, which makes the containment problem more challenging but practical and prospective.

Recently, output containment control (OCC) has attracted considerable attention for heterogeneous multi-agent systems with state variables of different dimensions. Using the internal model principle, OCC of heterogeneous multi-agent systems was studied under both state and output feedback designs in [9]. OCC of heterogeneous multi-agent systems was investigated using an output regulation technique by designing an optimally distributed PID-like controller in [10]. Specific limitations or task requirements, such as transmission delays [11], fixed-time [12], and input saturation [13], have been reported for OCC of heterogeneous multi-agent systems in recent literature. For more complex environments or certain specific task scenarios, bipartite formation-containment was studied for heterogeneous multi-agent systems in [14]. In [15], formation-containment control was investigated for heterogeneous linear multi-agent systems with unbounded transmission delays. In general, by applying the output regulation technique, a distributed observer or internal model is introduced to estimate the leader’s signal for each heterogeneous follower. Thus, the abovementioned studies generally require each follower to know the matrix *S* of the leader system [10,11,12,13,14,15], which may be unrealistic in some situations. In case of the unknown system matrix, some effective adaptive control methods, such as the leaning algorithms [16,17] and the adaptive estimation [18,19,20], have recently been developed. In [18], an adaptive approach was proposed to estimate the system matrix for each follower using a distributed adaptive estimation technique. In the case with multiple leaders, an adaptive distributed observer was designed to achieve the OCC of a multi-agent system in [19], in which only the system matrix *S* was estimated. To know the system matrices of the leaders, a novel adaptive OCC was studied under both state-feedback and dynamic output-feedback in [20]. However, few studies have considered the OCC of heterogeneous multi-agent systems over clustered networks. Thus, in this study, we investigate the adaptive OCC problem over clustered networks, particularly over more complex wide-area networks.

In general, complex networks in real-world applications may comprise several smaller subnetworks, such as the post-disaster emergency communication networks in [21]. Therefore, the investigation of the synchronization of wide-area networks is important. Intuitively, wide-area networks exhibit more complex phenomena than a simple network pattern due to task requirements or wide-area scenarios. Consequently, increasing attention has recently been paid to various control problems. A consensus control problem was investigated for clustered networks with impulsive communication in [22]. Furthermore, a static output feedback control was considered in [23] to achieve consensus. In [24], output consensus of clustered networks was achieved using a reduced-order observer. Subsequently, the work was extended to heterogeneous clustered networks to achieve output consensus in [25]. In the case of inter-cluster intermittent communication, an intermittent output tracking control was proposed for heterogeneous multi-agent systems over clustered networks in [26]. However, few studies have focused on clustered networks with multiple leaders, which inspired us to address the OCC problem for clustered networks.

Due to limited resources, physical device failures, and communication barriers, distributed intermittent control is desired owing to its effective and economical communication mode. Periodic [27,28,29] and aperiodic [30,31,32] intermittent control have been reported for multi-agent systems. To achieve containment in the case of periodic intermittent communication, intermittent containment control was investigated for second-order multi-agent systems in [33]. In [34], periodic intermittent containment control was explored for nonlinear multi-agent systems subjected to unknown disturbances. This control strategy was also extended to heterogeneous multi-agent systems in [35]. In contrast to periodic intermittent control, aperiodic intermittent control, which consists of aperiodic time intervals, is more realistic. For example, the wind power in [36] suffered from unstable wind speed. Considering time-delay and aperiodic intermittent communication, second-order multi-agent systems were exponentially stabilized utilizing a distributed aperiodic intermittent control strategy in [37]. In [38], a novel distributed aperiodic intermittent communication scheme was proposed for linear multi-agent systems with disturbances. By introducing time-scale theory, aperiodic intermittent containment control was investigated for a heterogeneous multi-agent system in [39]. However, the sum of the communication and non-communication lengths is required for the exponential stability in the abovementioned aperiodic intermittent control methods. To relax this strict constraint, a simple but practical condition is desired for distributed aperiodic intermittent controls.

Motivated by the above discussion, in this study, we propose a distributed aperiodic intermittent control approach to solve the output containment problem for a heterogeneous multi-agent system, without expecting all followers to know the system matrix *S* of the leader. To this end, a distributed adaptive observer is used to estimate the matrix *S*. Based on this, an adaptive distributed hybrid controller is designed using a dynamic compensator. Using a Lyapunov function method and the output regulation technique, sufficient conditions for the adaptive intermittent OCC are derived. The main contributions of this paper are as follows:

- A distributed adaptive approach is designed for the internal leaders to estimate the system matrix *S* of the homogeneous exogenous leaders. Compared with [37,38,39], this approach is more practical and extends to a wide-area network.
- Distributed hybrid controllers are designed separately for the internal leaders and followers to achieve output containment tracking. Specifically, the distributed aperiodic intermittent controller is designed for the internal leader, whereas the continuous dynamic feedback controller is designed for the follower based on the internal model.
- Sufficient conditions for the exponential stability of the closed-loop system are derived, where intermittent control rate and control parameters are calculated based on the average dwell-time and regulator equations.

The remainder of this paper is organized as follows. Section 2 presents essential preliminaries and formulates the problem statement and framework. The main results of the developed hybrid control algorithms and theories are presented in Section 3. Simulation examples are provided in Section 4. Finally, conclusions are presented in Section 5.

## 2. Preliminaries and Problem Formulation

In this section, we first introduce the basics of directed graph topology and the study problem based on a clustered hybrid communication network.

### 2.1. Notations

$R^{m\times n}$ denotes the set of m×n real matrices. $1_{n}$ and $I_{n}$ denote the column vector with *n* elements as 1 and the n−dimensional identity matrix, respectively. ⊗ is the Kronecker product. P>0(P<0) denotes a positive (negative) definite matrix. The induced 2-norm of the matrix or Euclidean vector norm is denoted as ∥•∥. Let $\lambda_{\max}\left(•\right)$ and $\lambda_{\min}\left(•\right)$ be the maximum and minimum eigenvalues, respectively. $\inf\{\tau_{i}\}$ and $\sup\{\tau_{i}\}$ denote the largest lower and smallest upper bounds of the set $\left\{\tau_{i}\right\}$, respectively. $\mathrm{diag}\{A_{1},A_{2},\cdots ,A_{n}\}$ denote a block-diagonal matrix with arbitrary matrices $A_{i}\in R^{m\times m},i=1,\cdots ,n$. For any column vector $\zeta =\mathrm{col}\left(w_{1},w_{2},\cdots ,w_{q}\right)\in R^{qn}$ with any vector $w_{i}\in R^{n}$, we define $M_{n}^{q}\left(\zeta \right)=\left[w_{1},w_{2},\cdots ,w_{q}\right]$. $\mathrm{dist}\left(y_{i},C\right)=\mathrm{in}f_{y_{0}\in C}{∥y_{i}-y_{0}∥}_{2}$ denotes the Euclidean distance from $y_{i}\in R^{n}$ to a set $C\subseteq R^{n}$.

**Definition 1.**  *Define $C\subseteq R^{n}$. For any $y_{i},y_{j}\in C$ and any λ∈[0,1], the set C is convex if $\left(1-\lambda \right)y_{i}+\lambda y_{j}\in C$. A convex hull, denoted as Co(Y), is the minimal convex set containing all points in $Y=\{y_{1},y_{2},\cdots ,y_{M_{0}}\}$, that is, $Co\left(Y\right)=\left\{\sum_{r=1}^{M_{0}}\alpha_{r}y_{r}|y_{r}\in Y,\alpha_{r}\geq 0,\sum_{r=1}^{M_{0}}\alpha_{r}=1\right\}$.*

### 2.2. Communication Network Modeling

A directed graph G=(V,E,A) is typically used to describe the communication network for a group of autonomous agents, where the node set $V=\{\upsilon_{1},\upsilon_{2},\cdots ,\upsilon_{N}\}$ denotes the set of *N* agents. The edge set $E=\{\left(\upsilon_{i},\upsilon_{j}\right):\;i,j\in V\}$ denotes the set of communication links between agents. A directed edge $\left(\upsilon_{i},\upsilon_{j}\right)$ implies that agent $\upsilon_{i}$ can receive information from neighboring agent $\upsilon_{j}$. Meanwhile, if $(\upsilon_{i},\upsilon_{j})\in E$, the weight is defined as $a_{ij}=1$; otherwise, $a_{ij}=0$. Thus, $A=\left[a_{ij}\right]\in R^{N\times N}$ is the adjacency matrix associated with the directed graph G. Additionally, the Laplacian matrix of the graph G is defined as $L=\left[l_{ij}\right]\in R^{N\times N}$, where $l_{ii}=\sum_{j=1,i\neq j}^{N}a_{ij}$ and $l_{ij}=-a_{ij}\left(i\neq j\right)$.

Regarding multi-area scenarios, the communication network G is spilt into *M* subnetworks described as $G_{k}=\left(V_{k},E_{k},A_{k}\right)\;\left(k=1,2,\cdots ,M\right)$, which are called clusters in sequence. It is assumed that each subnetwork has a spanning tree with $N_{k}$ followers and an internal leader indexed as $l_{k}$ as its root. Thus, *N* followers belong to the sets $V=\{V_{1},V_{2},\cdots ,V_{M}\}$ with $\partial_{k-1}=\sum_{j=0}^{k-1}N_{j}\;\left(\partial_{0}=0\right)$. The clusters satisfy the following properties: $V_{k}\neq \emptyset$, ${⋃}_{v=1}^{M}V_{k}=V$, and $V_{v}⋂V_{k}=\emptyset$(v≠k). In addition, for each cluster $G_{k}$, we define a Laplacian matrix $L_{k}=\left[l_{ij}\right]\in R^{N_{k}\times N_{k}}$ and a leader adjacency matrix $W_{k}=\mathrm{diag}\left(\omega_{\partial_{k-1}+1,l_{k}},\cdots ,\omega_{\partial_{k-1}+N_{k},l_{k}}\right)$, where $\omega_{\partial_{k-1}+ı,l_{k}}=1\;\left(ı=1,2,\cdots ,N_{k}\right)$ if $\varepsilon_{\partial_{k-1}+ı,l_{k}}\in E$, and $\omega_{\partial_{k-1}+ı,l_{k}}=0$ if $\varepsilon_{\partial_{k-1}+ı,l_{k}}\notin E$. Thus, the global interaction of all agents in $G_{k}$ is denoted as $H_{k}=L_{k}+W_{k}$.

As mentioned above, because communication among clusters is only implemented by *M* internal leaders, let $G_{L}$ be a directed graph to describe the communication network associated with the above leaders, which are indexed as $l_{k}\;\left(L_{M}=\left\{l_{1},\cdots ,l_{M}\right\}\right)$. Similarly, $L_{int}=\left[l_{l_{k}l_{v}}\right]\in R^{M\times M}$ is obtained for leaders. The objective of this paper is to drive the internal leaders to move into a desired region spanned by $M_{0}$ exogenous leaders, which are indexed as $l_{r}^{0},L_{M_{0}}=\left\{l_{1}^{0},\cdots ,l_{M_{0}}^{0}\right\}$. Thus, the interaction relationships of these $\bar{M}=M+M_{0}$ agents can be modeled using a directed graph ${\bar{G}}_{L}$. We define the adjacency matrix for each leader as $W_{r}=\mathrm{diag}\left(\omega_{l_{1}l_{r}^{0}},\omega_{l_{2}l_{r}^{0}},\cdots ,\omega_{l_{M}l_{r}^{0}}\right)$, where $\omega_{l_{k}l_{r}^{0}}=1$, if the leader $l_{k}$ is connected to the exogenous leader $l_{r}^{0}$; otherwise, $\omega_{l_{k}l_{r}^{0}}=0$. Combining $L_{\mathrm{int}}$ and $W_{r}$, we can define $H_{r}=\frac{1}{M_{0}}L_{\mathrm{int}}+W_{r}$. The necessary assumptions regarding the above descriptions are introduced below.

**Assumption 1.**  *There exists at least one directed path from the internal leader $l_{r}^{0}\;\left(l_{k}\right)$ to followers in the same cluster.*

**Figure 1:**
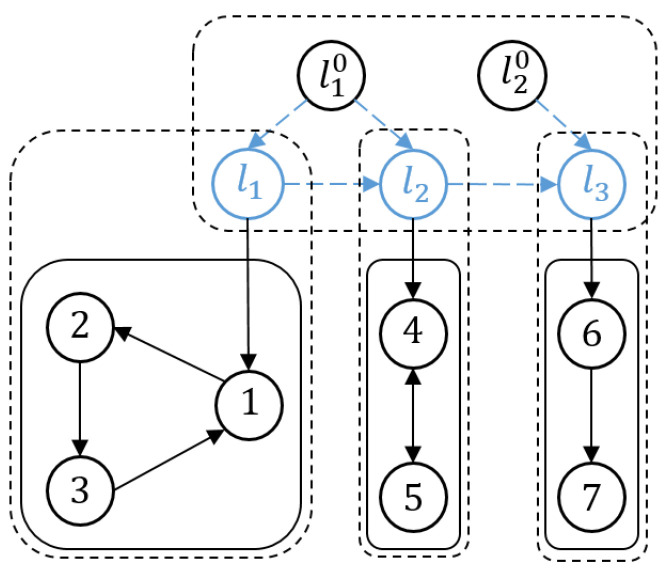
Wide-area network framework with hybrid communication.

Figure 1 illustrates the connectivity of the communication network associated with the followers i(i=1,⋯,7), internal leaders $l_{1},l_{2}$, and exogenous leaders $l_{1}^{0},l_{2}^{0}$.

**Lemma 1**  ([10]). *Under Assumption 1, the matrix $H_{r}=\frac{1}{M_{0}}L_{int}+W_{r}$ is invertible. A square matrix A is stochastic if all of its entries are non-negative and the entries of each row add up to 1.*

**Lemma 2**  ([40]). *Under Assumption 1, for M-matrix $\bar{H}=\sum_{r=1}^{M_{0}}H_{r}$, there exists a matrix $\bar{\Xi }=\mathrm{diag}\left(\delta_{l_{1}},\delta_{l_{2}},\cdots ,\delta_{l_{M}}\right)$ with positive scalar $\delta_{l_{k}}>0$ satisfying ${\left(\delta_{l_{1}},\delta_{l_{2}},\cdots ,\delta_{l_{M}}\right)}^{T}={\bar{H}}^{-T}1_{M}$, such that $\bar{\Xi }\bar{H}+{\bar{H}}^{T}\bar{\Xi }>0$. Similar to $\bar{H}$, a corresponding $\Xi_{k}$ exists for each $G_{k}$, satisfying $\Xi_{k}H_{k}+H_{k}^{T}\Xi_{k}>0$.*

### 2.3. Problem Statement

Consider a general wide-area communication network, where there are *N* heterogeneous followers and $M_{0}$ exogenous leaders with the following dynamics.

(1) $$\left\{\begin{array}{lll} {\dot{x}}_{i}\left(t\right)=A_{i}x_{i}\left(t\right)+B_{i}u_{i}\left(t\right), & \; & i\in G_{k}\\ y_{i}\left(t\right)=C_{i}x_{i}\left(t\right), \end{array}\right.$$

and

(2) $$\left\{\begin{array}{lll} {\dot{x}}_{l_{r}^{0}}\left(t\right)=Sx_{l_{r}^{0}}\left(t\right), & \; & r\in L_{M_{0}}\\ y_{l_{r}^{0}}\left(t\right)=Dx_{l_{r}}\left(t\right), \end{array}\right.$$

where $x_{i}\left(t\right)\in R^{n_{i}}$, $u_{i}\left(t\right)\in R^{m_{i}}$, and $y_{i}\left(t\right)\in R^{p}$ are the state, control input, and output of the *i*th follower, respectively. Similarly, the state, input, and output of the $l_{r}^{0}$th leader are denoted as $x_{l_{r}^{0}}\left(t\right)\in R^{n_{0}}$, $u_{l_{r}^{0}}\left(t\right)\in R^{m_{0}}$, and $y_{l_{r}^{0}}\left(t\right)\in R^{p}$, respectively. The constant real matrices $A_{i}\in R^{n_{i}\times n_{i}}$, $B_{i}\in R^{n_{i}\times m_{i}}$, $C_{i}\in R^{p\times n_{i}}$, $S\in R^{n_{0}\times n_{0}}$, and $D\in R^{p\times n_{0}}$.

Motivated by [18], the system matrix *S* of exogenous leaders may be unknown to other agents. By introducing an internal model and adaptive control method, the dynamics of $M_{1}$ internal leaders are modeled as

(3) $$\left\{\begin{array}{lll} {\dot{x}}_{l_{k}}\left(t\right)=S_{l_{k}}\left(t\right)x_{l_{k}}\left(t\right)+u_{l_{k}}\left(t\right), & \; & k\in L_{M}\\ y_{l_{k}}\left(t\right)=Dx_{l_{k}}\left(t\right), \end{array}\right.$$

where $x_{l_{k}}\left(t\right)\in R^{n_{0}}$, $u_{l_{k}}\left(t\right)\in R^{m_{0}}$, $y_{l_{k}}\left(t\right)\in R^{p}$, and $S_{l_{k}}\left(t\right)$ denote the state, input, output, and the estimation of *S*, respectively. In addition, the following assumptions are necessary.

**Assumption 2.**  *The pairs $\left(A_{i},B_{i}\right)$ and $\left(A_{i},C_{i}\right)$ are stabilizable and detectable, respectively.*

**Assumption 3.**  *The real parts of all eigenvalues of S are positive.*

**Assumption 4.**  *There exist the following matrix equations with corresponding solution pairs $\left(\Pi_{i},U_{i}\right)\;\left(i=1,2,\cdots ,N\right)$.*

(4) $$\left\{\begin{array}{l} \Pi_{i}S=A_{i}\Pi_{i}+B_{i}U_{i},\\ C_{i}\Pi_{i}=D. \end{array}\right.$$

For the case with multiple leaders in this study, we investigate the output containment problem of multi-area networks, which can be described in detail as follows.

**Definition 2.**  *The heterogeneous multi-agent system (1)–(3) over the clustered network can achieve output containment if for all general initial states, all followers’ outputs converge to the desired convex hull formed by the exogenous leaders as time t tends to infinity, that is,*

(5) $$\left\{\begin{array}{lll} \lim_{t\to \infty }\mathrm{dist}\left(y_{l_{k}}\left(t\right),Co\left(Y\left(t\right)\right)\right)=0, & \; & k\in L_{M}\\ \lim_{t\to \infty }\left(y_{i}\left(t\right)-y_{l_{k}}\left(t\right)\right)=0. & \; & i\in G_{k} \end{array}\right.$$

## 3. Main Results

In this section, by proposing an adaptive distributed intermittent control strategy, sufficient conditions for output containment are derived using the output feedback.

### 3.1. Distributed Hybrid Adaptive Control Strategy

**Figure 2:**
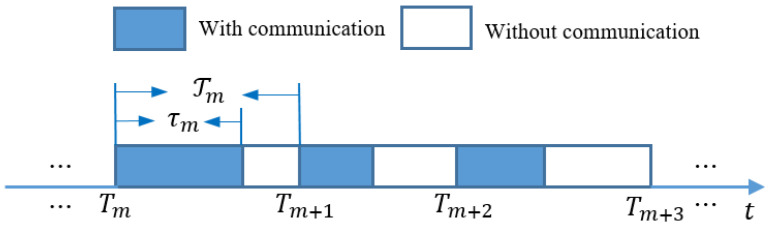
Aperiodically intermittent communication structure.

An aperiodic intermittent control mechanism is introduced because of the inevitable intermittent communication. Based on a non-periodic time sequence, the framework of aperiodic intermittent communication is intuitively developed and illustrated in Figure 2.

In this framework, it is clearly shown that $0=T_{0}<T_{1}<\cdots <T_{m}<\cdots$ such that the aperiodic time sequence can be denoted as ${\left\{T_{m}\right\}}_{m=0}^{\infty }$. There exists an aperiodic time interval $\tau_{m}$ in the non-periodic period $T_{m}=T_{m+1}-T_{m}$ satisfying $0<\tau_{m}<T_{m}$. This implies that each aperiodic intermittent period $\left[T_{m},T_{m+1}\right)$ is composed of two parts: $\left[T_{m},T_{m}+\tau_{m}\right]$ and $\left(T_{m}+\tau_{m},T_{m+1}\right)$. In general, the time widths, $\left[T_{m},T_{m}+\tau_{m}\right]$ with communication and $\left(T_{m}+\tau_{m},T_{m+1}\right)$ without communication, are also referred to as the work and rest time intervals, respectively. In addition, continuous communication is considered in each cluster $G_{k}$. By introducing this aperiodic intermittent control method, the global heterogeneous clustered network can be described as follows:

$$\begin{array}{l} \left\{\begin{array}{lll} \bar{H}\left(t\right)=\bar{H}, & \; & t\in [T_{m},T_{m}+\tau_{m}]\\ \bar{H}\left(t\right)=0, & \; & t\in (T_{m}+\tau_{m},T_{m+1})\\ H_{k}\left(t\right)=H_{k}. & \; & t\in [T_{m},T_{m+1}) \end{array}\right. \end{array}$$

**Assumption 5.**  *Based on the aperiodic intermittent control mechanism, there exist scalars $\gamma_{1}>0$, $\gamma_{2}>0$, and h>0 such that the following average intermittent intervals are defined as follows:*

$$\begin{array}{l} \varrho_{1}≜\lim_{m\to \infty }\inf\frac{\sum_{s=0}^{m}\tau_{s}}{m+1},\;\varrho_{2}≜\lim_{m\to \infty }\sup\frac{\sum_{s=0}^{m}\left(T_{s+1}-T_{s}\right)}{m+1},\;h≜\lim_{m\to \infty }\sup\frac{\sum_{s=0}^{m}T_{s+1}-T_{s}-\tau_{s}}{m+1}, \end{array}$$

satisfying $m\geq m^{*}\geq 1$, $\varrho_{2}>\varrho_{1}>0$, and $\varrho_{2}>h>0$.

**Remark 1.**  *According to the abovementioned description of aperiodic intermittent communication, the average time intervals $\varrho_{1}$, $\varrho_{2}$, and h can be observed and defined based on the time-scale theory. Each communication period comprises a pair of work and rest time intervals, which implies that $\varrho_{2}\geq \varrho_{1}>0$ over the time sequence ${\left\{T_{m}\right\}}_{m=0}^{\infty }$, and $\varrho_{2}>h>0$ is obtained for the aperiodic intermittent communication. Under Assumption 5, a novel criterion for intermittent control can be derived, in which the intermittent rate is related to the defined average time intervals. Additionally, in Assumption 1, the leader is the root of the spanning tree, which ensures that the Laplacian matrix $L_{int}\left(L_{k}\right)$ has no eigenvalues with negative real-parts for each clustered network $G_{L}\left(G_{K}\right)$. Assumption 2 is used to guarantee the existence of the gain matrices such that the closed-loop system is stable and the observer is convergent. Under Assumption 3, the exogenous signals can be unbounded, which is more challenging than the cases when the matrix S has zero eigenvalues or eigenvalues with negative real-parts. Assumption 4 is the standard condition for the solvability of the linear output regulation problems.*

To achieve containment tracking over the clustered network, we design a distributed hybrid control for the heterogeneous multi-agent system. Specifically, a distributed intermittent controller is proposed for the internal leader $l_{k}$ as follows:

(6) $$\left\{\begin{array}{l} u_{l_{k}}\left(t\right)\;=\;d\;\left(\sum_{v\in L_{M}}a_{l_{k}l_{v}}\left(x_{l_{v}}\left(t\right)\;-\;x_{l_{k}}\left(t\right)\right)\;+\;\sum_{r\in L_{M_{0}}}\omega_{l_{k}l_{r}^{0}}\left(x_{l_{r}^{0}}\left(t\right)\;-\;x_{l_{k}}\left(t\right)\right)\;\right),\;t\;\in \;\left[T_{m},T_{m}\;+\;\tau_{m}\right]\\ u_{l_{k}}\left(t\right)\;=\;0,\;t\in \left(T_{m}+\tau_{m},T_{m+1}\right) \end{array}\right.$$

where the control gain *d* is an arbitrary positive constant.

To estimate the system matrix *S* of the exogenous leader, we design distributed adaptive estimation laws for the internal leaders and followers as follows:

(7) $$\left\{\begin{array}{l} {\dot{S}}_{l_{k}}\left(t\right)=\gamma_{1}\left(\sum_{v\in L_{M}}a_{l_{k}l_{v}}\left(S_{l_{v}}\left(t\right)-S_{l_{k}}\left(t\right)\right)\right)+\sum_{r\in L_{M_{0}}}\omega_{l_{k}l_{r}^{0}}\left(S-S_{l_{k}}\left(t\right)\right),\\ {\dot{S}}_{i}\left(t\right)=\gamma_{2}\left(\sum_{j\in G_{k}}a_{ij}\left(S_{j}\left(t\right)-S_{i}\left(t\right)\right)+\omega_{il_{k}}\left(S_{l_{k}}\left(t\right)-S_{i}\left(t\right)\right)\right), \end{array}\right.$$

where $S_{i}\left(t\right)$ and $S_{l_{k}}\left(t\right)$ denote the estimation of the system matrix *S*, $\gamma_{1}$ and $\gamma_{2}>0$ are positive constants.

By introducing the compensator technique, an adaptive distributed hybrid controller with output feedback design for each heterogeneous follower takes the following form:

(8) $$\left\{\begin{array}{l} u_{i}\left(t\right)=K_{1i}{\hat{x}}_{i}\left(t\right)+K_{2i}\left(t\right)z_{i}\left(t\right),\;i\in G_{k}\\ {\dot{\hat{x}}}_{i}\left(t\right)=A_{i}{\hat{x}}_{i}\left(t\right)+B_{i}u_{i}\left(t\right)-F_{i}C_{i}\left(x_{i}\left(t\right)-{\hat{x}}_{i}\left(t\right)\right),\\ {\dot{z}}_{i}\left(t\right)=S_{i}\left(t\right)z_{i}\left(t\right)+d\left(\sum_{j\in G_{k}}a_{ij}\left(z_{j}\left(t\right)-z_{i}\left(t\right)\right)+\omega_{il_{k}}\left(x_{l_{k}}\left(t\right)-z_{i}\left(t\right)\right)\right),\;t\in \left[T_{m},T_{m+1}\right) \end{array}\right.$$

where ${\hat{x}}_{i}\left(t\right)$ denotes the estimation of $x_{i}\left(t\right)$, $z_{i}\left(t\right)\in R^{n_{0}}$ represents the state of the *i*th internal model, $K_{1i}$, $K_{2i}\left(t\right)$, and $F_{i}$ are the control gains.

### 3.2. Error System Modeling

To analyze the convergence of the adaptive estimation laws (7), two error variables are defined as follows:

(9) $$\left\{\begin{array}{l} {\tilde{S}}_{l_{k}}\left(t\right)=S_{l_{k}}\left(t\right)-S,\\ {\tilde{S}}_{il_{k}}\left(t\right)=S_{i}\left(t\right)-S_{l_{k}}\left(t\right),\;i\in G_{k} \end{array}\right.$$

Furthermore, for k=1,2,⋯,M, we define $S_{L}\left(t\right)={\left[{\tilde{S}}_{l_{1}}^{T}\left(t\right),{\tilde{S}}_{l_{2}}^{T}\left(t\right),\cdots ,{\tilde{S}}_{l_{M}}^{T}\left(t\right)\right]}^{T}$ and $S_{k}\left(t\right)={\left[{\tilde{S}}_{\partial_{r-1}+1,l_{k}}^{T}\left(t\right),{\tilde{S}}_{\partial_{r-1}+2,l_{k}}^{T}\left(t\right),\cdots ,{\tilde{S}}_{\partial_{r-1}+N_{k},l_{k}}^{T}\left(t\right)\right]}^{T}$. Define $\bar{H}={\left[{\bar{H}}_{1L}^{T},\cdots ,{\bar{H}}_{kL}^{T},\cdots ,{\bar{H}}_{ML}^{T}\right]}^{T}$ and ${\bar{H}}_{k}=1_{N_{k}}⊗{\bar{H}}_{kL}$. From (7), it follows that

(10) $$\left\{\begin{array}{l} {\dot{S}}_{L}\left(t\right)=-\gamma_{1}\left(\bar{H}⊗I_{n_{0}}\right)S_{L}\left(t\right),\\ {\dot{S}}_{k}\left(t\right)=-\gamma_{2}\left(H_{k}⊗I_{n_{0}}\right)S_{k}\left(t\right)+\gamma_{1}\left({\bar{H}}_{k}⊗I_{n_{0}}\right)S_{L}\left(t\right). \end{array}\right.$$

To estimate the states of the leaders, a second form of the error variables is defined as follows:

(11) $$\left\{\begin{array}{l} e_{l_{k}l_{r}^{0}}\left(t\right)=x_{l_{k}}\left(t\right)-x_{l_{r}^{0}}\left(t\right),\\ e_{il_{k}}\left(t\right)=z_{i}\left(t\right)-x_{l_{k}}\left(t\right).\;i\in G_{k} \end{array}\right.$$

Regarding the leader-following tracking, we define the following error for each internal leader:

(12) $$\begin{array}{l} e_{l_{k}}\left(t\right)=\sum_{v\in L_{M}}a_{l_{k}l_{v}}\left(x_{l_{v}}\left(t\right)-x_{l_{k}}\left(t\right)\right)+\sum_{r\in L_{M_{0}}}\omega_{l_{k}l_{r}^{0}}\left(x_{l_{r}^{0}}\left(t\right)-x_{l_{k}}\left(t\right)\right). \end{array}$$

Moreover, we define $e_{L}\left(t\right)={\left[e_{l_{1}}^{T}\left(t\right),e_{l_{2}}^{T}\left(t\right),\cdots ,e_{l_{M}}^{T}\left(t\right)\right]}^{T}\in R^{Mn_{0}}$, $x_{L}\left(t\right)={\left[x_{l_{1}}^{T}\left(t\right),x_{l_{2}}^{T}\left(t\right),\cdots ,x_{l_{M}}^{T}\left(t\right)\right]}^{T}\in R^{Mn_{0}}$, and ${\bar{x}}_{r}\left(t\right)=1_{M}⊗x_{l_{r}^{0}}\left(t\right)$. Next, we rewrite $e_{L}\left(t\right)$ as $e_{L}\left(t\right)=-\sum_{r\in L_{M_{0}}}\left(H_{r}⊗I_{n_{0}}\right)\left(x_{L}\left(t\right)-{\bar{x}}_{r}\left(t\right)\right)$. Given $\phi \left(t\right)=-\sum_{r\in L_{M_{0}}}{\left(H_{r}⊗I_{n_{0}}\right)}^{-1}e_{L}\left(t\right)$, we obtain

(13) $$\begin{array}{l} \phi \left(t\right)=x_{L}\left(t\right)-{\left(\sum_{\bar{r}\in L_{M_{0}}}\left(H_{\bar{r}}⊗I_{n_{0}}\right)\right)}^{-1}\sum_{r\in L_{M_{0}}}\left(H_{r}⊗I_{n_{0}}\right){\bar{x}}_{r}\left(t\right). \end{array}$$

Defining $\phi \left(t\right)=x_{L}\left(t\right)-\Phi \left(t\right)$ and $\Phi \left(t\right)={\left(\sum_{\bar{r}\in L_{M_{0}}}\left(H_{\bar{r}}⊗I_{n_{0}}\right)\right)}^{-1}\sum_{r\in L_{M_{0}}}\left(H_{r}⊗I_{n_{0}}\right)$${\bar{x}}_{r}\left(t\right)$ implies that $\dot{\Phi }\left(t\right)=\left(I_{N}⊗S\right)\Phi \left(t\right)$, according to the leader’s dynamics described by (2). Since ${\tilde{S}}_{l_{k}}\left(t\right)=S_{l_{k}}\left(t\right)-S$, we define $\hat{S}\left(t\right)=\mathrm{block}\;\mathrm{diag}\left\{{\tilde{S}}_{l_{1}}\left(t\right),{\tilde{S}}_{l_{2}}\left(t\right),\cdots ,{\tilde{S}}_{l_{M}}\left(t\right)\right\}$ for convenience. Combining (2), (6), and (7), the dynamics of ϕ(t) under the intermittent control can be expressed as follows:

(14) $$\left\{\begin{array}{ll} \dot{\phi }\left(t\right) & =\hat{S}\left(t\right)\phi \left(t\right)+\hat{S}\left(t\right)\Phi \left(t\right)+\left(I_{N}⊗S-d\bar{H}⊗I_{n_{0}}\right)\phi \left(t\right),\;t\in \left[T_{m},T_{m}+\tau_{m}\right]\\ \dot{\phi }\left(t\right) & =\hat{S}\left(t\right)\phi \left(t\right)+\hat{S}\left(t\right)\Phi \left(t\right)+\left(I_{N}⊗S\right)\phi \left(t\right).\;t\in \left(T_{m}+\tau_{m},T_{m+1}\right) \end{array}\right.$$

Similarly, for $i\in G_{k}$, we define ${\hat{S}}_{k}\left(t\right)=\mathrm{block}\;\mathrm{diag}\left\{S_{l_{k}}\left(t\right),\cdots ,S_{l_{k}}\left(t\right)\right\}\in R^{N_{k}\times N_{k}}$, $e_{k}\left(t\right)={\left[e_{\partial_{r-1}+1,l_{k}}^{T}\left(t\right),e_{\partial_{r-1}+2,l_{k}}^{T}\left(t\right),\cdots ,e_{\partial_{r-1}+N_{k},l_{k}}^{T}\left(t\right)\right]}^{T}$, and $x_{k}\left(t\right)=1_{N_{k}}⊗x_{l_{k}}\left(t\right)$. Using (2), (7), and (8), the error system of the followers within each cluster $G_{k}$ is written as follows:

(15) $$\left\{\begin{array}{ll} {\dot{e}}_{k}\left(t\right)= & S_{k}\left(t\right)e_{k}\left(t\right)+S_{k}\left(t\right)x_{k}\left(t\right)-{\hat{S}}_{k}\left(t\right)e_{k}\left(t\right)\\ \; & -d\left(H_{k}⊗I_{n_{0}}\right)e_{k}\left(t\right)+{\bar{H}}_{k}e_{L}\left(t\right),\;t\in \left[T_{m},T_{m}+\tau_{m}\right]\\ {\dot{e}}_{k}\left(t\right)= & S_{k}\left(t\right)e_{k}\left(t\right)+S_{k}\left(t\right)x_{k}\left(t\right)-{\hat{S}}_{k}\left(t\right)e_{k}\left(t\right).\;t\in \left(T_{m}+\tau_{m},T_{m+1}\right) \end{array}\right.$$

### 3.3. Output Containment Analysis

**Lemma 3.**  *Under Assumptions 1 and 3, given the system (1) with an adaptive observer in (7), for any general initial states $S_{i}\left(0\right)$ and S(0), the trajectories of ${\tilde{S}}_{l_{k}}\left(t\right)$ and ${\tilde{S}}_{il_{k}}\left(t\right)$ are exponentially stable as t→∞.*

**Proof.**  According to Lemma 1, $\bar{H}$ is a non-negative *M*-matrix, which implies that all eigenvalues of $\bar{H}$ have positive real parts. Let $\mu_{0}=R\left(\sigma_{min}\left(\bar{H}\right)\right)>0$, then it follows from (10) that ${\dot{S}}_{L}\left(t\right)\leq -\gamma_{1}\mu_{0}S_{L}\left(t\right)$. That is,

(16) $$\begin{array}{l} ∥S_{L}\left(t\right)∥\;\leq \;∥S_{L}\left(0\right)∥e^{-\gamma_{1}\mu_{0}t}. \end{array}$$

As t→∞, this implies that $e^{-\gamma_{1}\mu_{0}t}\to 0$. Then, the error $\lim_{t\to \infty }{\tilde{S}}_{l_{k}}\left(t\right)=0$. □

Similarly, within each cluster from (10), by denoting $\mu_{k}=R\left(\sigma_{min}\left(H_{k}\right)\right)$ and ${\bar{\mu }}_{k}=\max\left\{R\left(\sigma_{\max}\left({\bar{H}}_{k}\right)\right)\right\}$, we obtain ${\dot{S}}_{k}\left(t\right)\leq -\gamma_{2}\mu_{k}S_{k}\left(t\right)+\gamma_{1}{\bar{\mu }}_{k}S_{L}\left(t\right)$. That is, $∥S_{k}\left(t\right)∥\;\leq \;∥S_{k}\left(0\right)∥e^{-\gamma_{2}\mu_{k}t}+∥\Lambda_{k}∥\int_{0}^{t}e^{-\gamma_{2}\mu_{k}\left(t-\tau \right)}e^{-\gamma_{1}\mu_{0}\tau }d\tau$ with $\Lambda_{k}=I_{N_{k}}⊗\gamma_{1}{\bar{\mu }}_{k}∥S_{L}\left(0\right)∥I_{n_{0}}$. Thus, we obtain

(17) $$∥S_{k}\left(t\right)∥\;\leq \left(∥S_{k}\left(0\right)∥-\frac{∥\Lambda_{k}∥}{\gamma_{2}\mu_{k}-\gamma_{1}\mu_{0}}\right)e^{-\gamma_{2}\mu_{k}t}+\frac{∥\Lambda_{k}∥}{\gamma_{2}\mu_{k}-\gamma_{1}\mu_{0}}e^{-\gamma_{1}\mu_{0}t}.$$

From (16) and (17), for $\gamma_{2}\mu_{k}\neq \gamma_{1}\mu_{0}$, we conclude that $e^{-\gamma_{2}\mu_{k}t}\to 0$ and $e^{-\gamma_{1}\mu_{0}t}\to 0$ as t→∞. That is, $\lim_{t\to \infty }{\tilde{S}}_{l_{k}}\left(t\right)=0$ and $\lim_{t\to \infty }{\tilde{S}}_{il_{k}}\left(t\right)=0$ are derived. Thus, the system matrix *S* can be estimated by all other agents using the proposed adaptive algorithm.

To achieve output containment, a feedforward control strategy is utilized as shown in (7), in which the control gain $K_{2i}$ is determined under the solution of regulator Equation (4). Because all followers do not know *S*, their estimate $S_{i}\left(t\right)$ is used to calculate the solution of (4) based on an adaptive control approach. Thus, using Lemma 1 in [41], the following lemma is derived.

**Lemma 4.**  *Under Assumptions 2–4, considering the multi-agent system (1)–(3), for any initial state $\zeta_{i}\left(0\right)$, we obtain the following equation:*

(18) $$\begin{array}{l} {\dot{\zeta }}_{i}\left(t\right)=-Q_{i}^{T}\left(t\right)\left(Q_{i}\left(t\right)\zeta_{i}\left(t\right)-b_{i}\right), \end{array}$$

*where $Q_{i}\left(t\right)=S_{i}^{T}\left(t\right)⊗\left[\begin{array}{cc} I_{n_{i}} & 0_{n_{i}\times m_{i}}\\ 0_{p\times n_{i}} & 0_{p\times m_{i}} \end{array}\right]-I_{n_{0}}⊗\left[\begin{array}{cc} A_{i} & B_{i}\\ C_{i} & D_{i} \end{array}\right]$, $b_{i}=vec\left(\left[\begin{array}{c} E_{i}\\ F_{i} \end{array}\right]\right)$, and $\zeta_{i}\left(t\right)=vec\left(\left[\begin{array}{c} \Pi_{i}\left(t\right)\\ U_{i}\left(t\right) \end{array}\right]\right)$, which have a unique solution if $\lim_{t\to \infty }\left(S_{i}\left(t\right)-S\right)=0$. Moreover, letting $M_{\left(n_{i}\times m_{i}\right)}^{q}\left(\zeta_{i}\left(t\right)\right)={\left[\Pi_{i}^{T}\left(t\right),U_{i}^{T}\left(t\right)\right]}^{T}$, we have*

(19) $$\begin{array}{l} \lim_{t\to \infty }\left(\left[\begin{array}{c} \Pi_{i}\left(t\right)\\ U_{i}\left(t\right) \end{array}\right]-\left[\begin{array}{c} \Pi_{i}^{*}\\ U_{i}^{*} \end{array}\right]\right)=0, \end{array}$$

where $\left(\Pi_{i}^{*},U_{i}^{*}\right)$ is the solution of the regulator Equation (4).

**Remark 2.**  In this paper, we extend the adaptive algorithm from the output regulation problem in [18] to the output containment problem over an intermittent communication network. Using Lemma 3, we achieve $\lim_{t\to \infty }\left(S_{i}\left(t\right)-S-\left(S_{l_{k}}\left(t\right)-S\right)\right)=0$ exponentially. This implies that the proposed adaptive observer can estimate S. Furthermore, using Lemma 4, it is easy to deduce that there exists a pair $\left(\Pi_{i}^{*},U_{i}^{*}\right)$, such that $\lim_{t\to \infty }\left(\left[\begin{array}{c} \Pi_{i}\left(t\right)\\ U_{i}\left(t\right) \end{array}\right]-\left[\begin{array}{c} \Pi_{i}^{*}\\ U_{i}^{*} \end{array}\right]\right)=0$. Meanwhile, the control gain $K_{2i}$ can be calculated based on the adaptive control method and regulator Equation (4). In this paper, we considered the adaptive containment tracking problem for heterogeneous multi-agent systems. Since the leaders’ dynamics can only be known to the neighboring agents, an adaptive algorithm has to be proposed to estimate the unknown system information for the other agents. It should be noted that the online reinforcement learning approach (or adaptive dynamic programming) [16] or policy iteration approach [17] were proposed to solve the optimal control of multi-agent systems with completely unknown system information. The optimal containment control of multi-agent systems with wide-area networks will be our future work.

Considering the underlying graph ${\bar{G}}_{L}$ with intermittent communication, sufficient conditions for the exponential stability of the switched system (14) are obtained using an aperiodic intermittent control method.

**Theorem 1.**  *Suppose that Assumptions 1, 3, and 5 are satisfied. Given the switched error system (14), $\lim_{t\to \infty }\phi \left(t\right)=0$ is achieved exponentially if the following conditions are satisfied:*

*(1) Given the appropriate matrices Q>0 and P>0, there exist scalars d>0, β>0, $\nu_{0}>0$, θ>0, and $\bar{\rho }>0$, such that*

(20) $$\left\{\begin{array}{l} PS+S^{T}P+\theta P+2\nu_{0}P+Q-d\bar{\rho }P<0,\\ PS+S^{T}P+\theta P+2\nu_{0}P-\beta P<0. \end{array}\right.$$

*(2) Given the appropriate scalars α>0 and σ>0, then the aperiodic intermittent rates $\frac{\varrho_{1}}{\varrho_{2}-\varrho_{1}}>\frac{\beta }{\alpha }$ and $\frac{\varrho_{1}}{\varrho_{2}-\varrho_{1}}>\frac{\beta }{\sigma }$.*

**Proof**  See Appendix A. □

**Remark 3.**  *Unlike [37,38,39], herein, Assumption 5 is applied for the aperiodic intermittent control mechanism. Therefore, the switched system (14) can be exponentially stabilized if (20) is feasible. In addition, the exponential convergence index is determined under the two appropriate intermittent rates derived from the inequality (A18). The stability of switched systems (14) not only relies on the control gain d, but also satisfies the intermittent rate $\frac{\varrho_{1}}{\varrho_{2}-\varrho_{1}}>\frac{\beta }{\alpha }$ under Assumption 5. Moreover, α>σ implies that $\frac{\varrho_{1}}{\varrho_{2}-\varrho_{1}}>\frac{\beta }{\sigma }$.*

**Remark 4.**  *Compared with the results in [37,38,39,42], herein, a novel stability criterion is derived for the aperiodic intermittent containment control, in which the two derived intermittent rates consist only of the average time intervals ${\bar{\tau }}_{1}$ and ${\bar{\tau }}_{2}$, and scalars α, β, and σ. Based on the developed intermittent rates, the final exponential convergence index is determined directly by comparison with [42]. Unlike [39], in this study, the current time sequence value m is removed from the obtained intermittent rates. The proposed aperiodic intermittent control method can be applied to various systems.*

Sufficient conditions for intermittent OCC under adaptive and intermittent control methods are then presented.

**Theorem 2.**  *Under Assumptions 1–5, considering the heterogeneous multi-agent system (1)–(3), leader-following output consensus is achieved within each cluster under the following conditions:*

*(1) Given appropriate scalars $\varepsilon_{1k}>0$, $\varepsilon_{2k}>0$, $\gamma_{2}>0$, $\mu_{k}>0$, and d>0, the following inequality holds:*

(21) $$\left\{\begin{array}{l} \gamma_{1}\mu_{0}-R\left(\sigma_{\max}\left(S\right)\right)>0,\\ \gamma_{2}\mu_{k}-R\left(\sigma_{\max}\left(S\right)\right)>0. \end{array}\right.$$

*(2) Given $A_{i}+B_{i}K_{1i}$ and $A_{i}+F_{i}C_{i}$ are Hurwitz matrices, let $K_{2i}\left(t\right)=U_{i}\left(t\right)-K_{1i}\Pi_{i}\left(t\right)$ with the solution $\left(U_{i}\left(t\right),\Pi_{i}\left(t\right)\right)$.*

**Proof**  See Appendix B. □

**Remark 5.**  *Because the states of heterogeneous agents cannot be obtained, an observer approach is utilized to estimate the state information under output feedback. In contrast to [19,20] with continuous communication, an adaptive distributed intermittent control strategy is proposed to estimate the output information and S of exogenous leaders. Although the proposed strategy is challenging, it is more realistic.*

**Remark 6.**  *In [41], it is assumed that the exogenous signal is bounded. To relax this constraint, Assumption 3 is applied in this study. Additionally, this assumption is necessary regarding the intermittent control scheme. To exponentially stabilize the switched error system (15), $\gamma_{1}\mu_{0}-R\left(\sigma_{\max}\left(S\right)\right)>0$ is derived, which implies that $\hat{S}\left(t\right)\Phi \left(t\right)$ exponentially decays to zero.*

## 4. Numerical Examples

**Figure 3:**
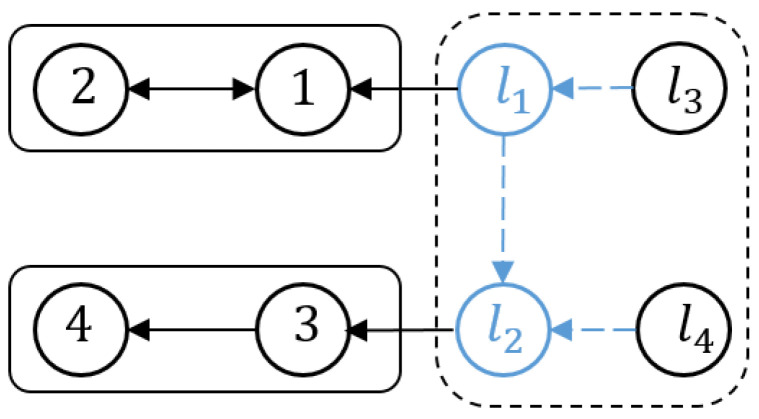
Heterogeneous intermittent communication network.

In this section, a simulation example is provided to demonstrate the effectiveness of the developed hybrid control methods for output containment. To simplify the description, we consider a complex network with four followers, two internal leaders, and two reference leaders. The intermittent communication network with the followers i(i=1,⋯,4), internal leaders $l_{1},l_{2}$, and exogenous leaders $l_{3},l_{4}$ is shown in Figure 3.

The dynamics of the system (1) with four heterogeneous followers are as follows:

$$\begin{array}{l} A_{1}=\left[\begin{array}{ccc} -1 & 1 & 0\\ 0 & 0 & 1\\ 0 & -1 & 0.5 \end{array}\right],B_{1}=\left[\begin{array}{cc} 0 & 0\\ 0 & 0\\ 1 & 0.2 \end{array}\right],C_{1}=\left[\begin{array}{ccc} 0 & 0 & 1 \end{array}\right].\\ A_{2}=\left[\begin{array}{ccc} -1 & 1 & 0\\ 0 & 0 & 1.5\\ 0 & -1 & 2 \end{array}\right],B_{2}=\left[\begin{array}{cc} 0 & 0\\ 0 & 0\\ 1 & 2 \end{array}\right],C_{2}=\left[\begin{array}{ccc} 0 & 0 & 1 \end{array}\right].\\ A_{3}=\left[\begin{array}{cc} 0 & 1\\ -2 & -0.8 \end{array}\right],B_{3}=\left[\begin{array}{c} 0\\ 1 \end{array}\right],C_{3}=\left[\begin{array}{cc} 0 & 1 \end{array}\right].\\ A_{4}=\left[\begin{array}{cc} 0 & 1\\ -1.5 & -1 \end{array}\right],B_{4}=\left[\begin{array}{c} 0\\ 1.2 \end{array}\right],C_{4}=\left[\begin{array}{cc} 0 & 1 \end{array}\right]. \end{array}$$

Moreover, the system matrices of leaders are given as follows:

$$\begin{array}{l} S=\left[\begin{array}{cc} 0 & 1\\ -1 & 0 \end{array}\right],D=\left[\begin{array}{cc} 1 & 0 \end{array}\right]. \end{array}$$

**Example 1.**  Based on Assumption 2, to illustrate the validity of Theorem 1, $K_{2i}\left(t\right)$ can be obtained using the adaptive regulator equations. Moreover, $K_{1i}$ and $K_{2i}^{*}$ are given as follows:

$$\begin{array}{l} K_{11}=\left[\begin{array}{ccc} -0.0410 & -0.7243 & -2.6574\\ -0.0082 & -0.1449 & -0.5315 \end{array}\right],K_{21}^{*}=\left[\begin{array}{cc} 2.6369 & -0.7448\\ -1.9726 & -0.1490 \end{array}\right]. \end{array}$$

$$\begin{array}{l} K_{12}=\left[\begin{array}{ccc} -0.0350 & -0.5246 & -1.6757\\ -0.0700 & -1.0493 & -3.3514 \end{array}\right],K_{22}^{*}=\left[\begin{array}{cc} 1.6582 & -0.5421\\ 2.3164 & -1.0843 \end{array}\right].\\ K_{13}=\left[\begin{array}{cc} 0.0004 & 0.0013 \end{array}\right],K_{23}^{*}=\left[\begin{array}{cc} 0.7987 & -0.9996 \end{array}\right].\\ K_{14}=\left[\begin{array}{cc} 0.0039 & 0.0104 \end{array}\right],K_{24}^{*}=\left[\begin{array}{cc} 0.8229 & -0.4127 \end{array}\right]. \end{array}$$

The gain matrices $F_{i}$ of the developed observers are given as follows:

$$\begin{array}{l} F_{1}=\left[\begin{array}{c} -0.8346\\ -0.3165\\ 1.3837 \end{array}\right],F_{2}=\left[\begin{array}{c} -0.6589\\ -0.2983\\ 3.4072 \end{array}\right],F_{3}=\left[\begin{array}{c} 0.0035\\ 0.0128 \end{array}\right],F_{4}=\left[\begin{array}{c} 0.0043\\ 0.0114 \end{array}\right]. \end{array}$$

We set parameters d=1, α=0.2, and β=0.3, and choose the matrix P>0 as

$$\begin{array}{l} P=\left[\begin{array}{cc} 1.1576 & -0.1373\\ -0.1373 & 1.1301 \end{array}\right]. \end{array}$$

From (A19), we set the aperiodic intermittent rate as $\frac{\varrho_{1}}{\varrho_{2}-\varrho_{1}}=\frac{\beta }{\sigma }\geq \frac{3}{2}$. Under Assumption 5, two time intervals are described as $\tau_{m}=0.6+0.1\;sin\left(t\right)$ and $T_{m+1}-T_{m}-\tau_{m}=0.4-0.1\;sin\left(t\right)$. To simplify the analysis, we choose the initial states $x_{i}\left(0\right)$, $z_{i}\left(0\right)$, and $x_{l_{r}}\left(0\right)$ within the interval [−1,1]. For ease of expression, the aperiodic intermittent control inputs of the internal leaders are expressed as follows:

$$\left\{\begin{array}{l} u_{l_{k}}\left(t\right)=d\left(\sum_{v\in L_{M}}a_{l_{k}l_{v}}\left(x_{l_{v}}\left(t\right)-x_{l_{k}}\left(t\right)\right)+\sum_{r\in L_{M_{0}}}\omega_{l_{k}l_{r}^{0}}\left(x_{l_{r}^{0}}\left(t\right)-x_{l_{k}}\left(t\right)\right)\right),\;t\in \left[T_{m},T_{m}+\tau_{m}\right]\\ u_{l_{k}}\left(t\right)=0,\;t\in \left(T_{m}+\tau_{m},T_{m+1}\right) \end{array}\right.$$

**Figure 4:**
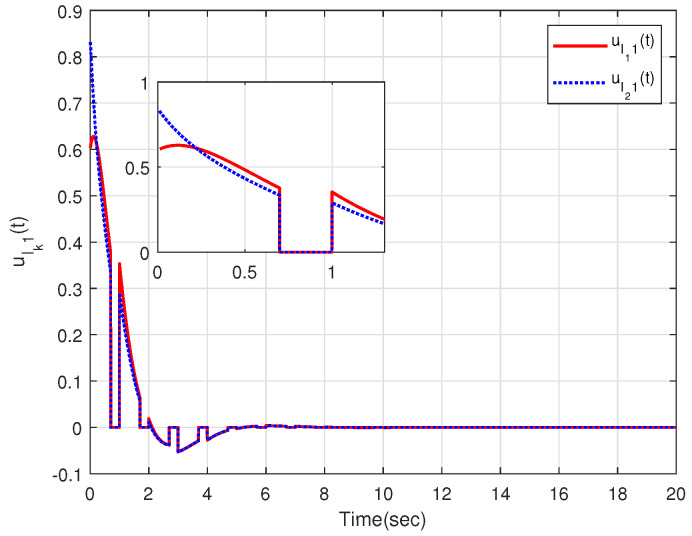
Intermittent control inputs $u_{l_{k}1}\left(t\right)$ of the internal leaders.

**Figure 5:**
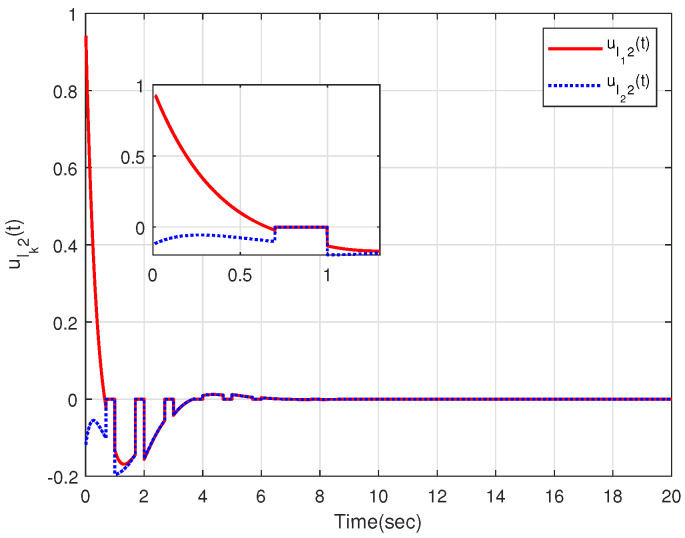
Intermittent control inputs $u_{l_{k}2}\left(t\right)$ of the internal leaders.

**Figure 6:**
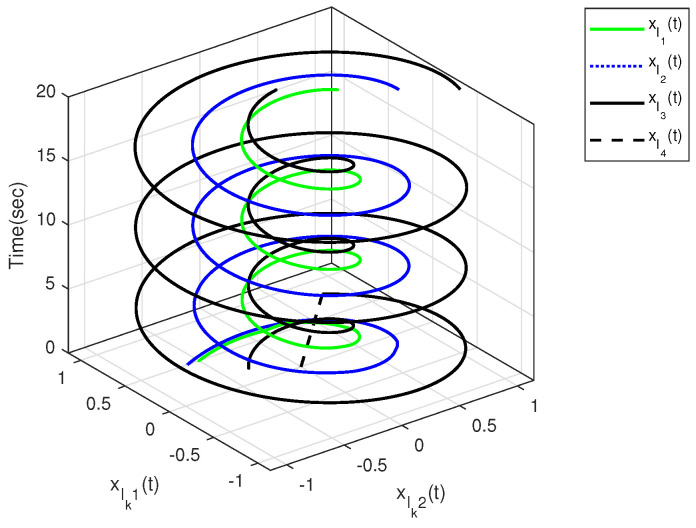
State trajectories $x_{l_{k}}\left(t\right)$ of all leaders.

The characteristic of aperiodic intermittent control input is intuitively reflected in Figure 4 and Figure 5, from which the control input converges to zero based on the developed distributed aperiodic intermittent control approach. That is, the designed intermittent rates under Assumption 5 are effective for aperiodic intermittent control. The state trajectories $x_{l_{k}}\left(t\right)$ are displayed in Figure 6, from which $x_{l_{1}}\left(t\right)$ and $x_{l_{2}}\left(t\right)$ enter the desired set spanned by leaders $x_{l_{3}}\left(t\right)$ and $x_{l_{4}}\left(t\right)$. That is, containment tracking is realized by the proposed adaptive distributed aperiodic intermittent control strategy.

**Figure 7:**
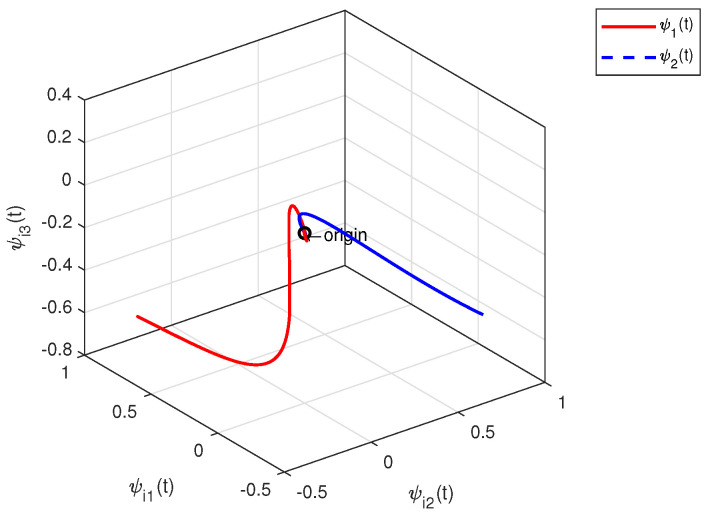
Observer error trajectories $\psi_{i}\left(t\right)$.

**Figure 8:**
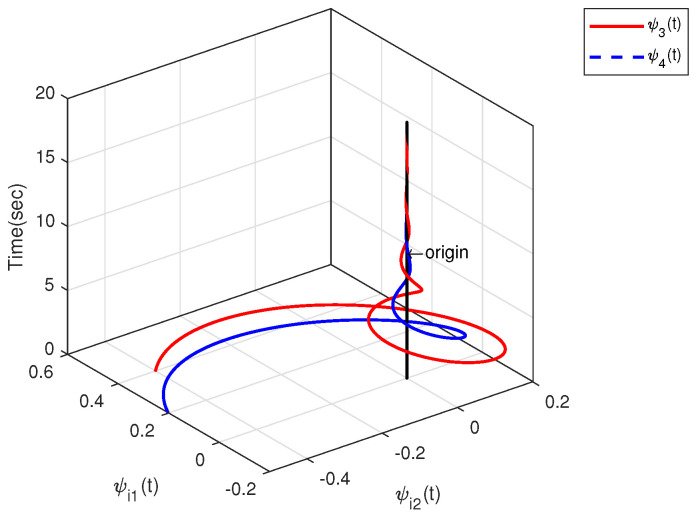
Observer error trajectories $\psi_{i}\left(t\right)$.

**Figure 9:**
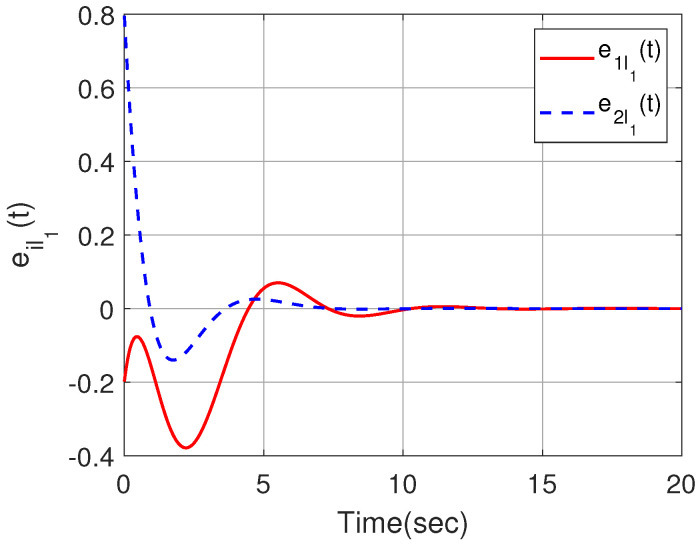
Output trajectories $e_{il_{1}}\left(t\right)$ within the cluster $G_{1}$.

**Figure 10:**
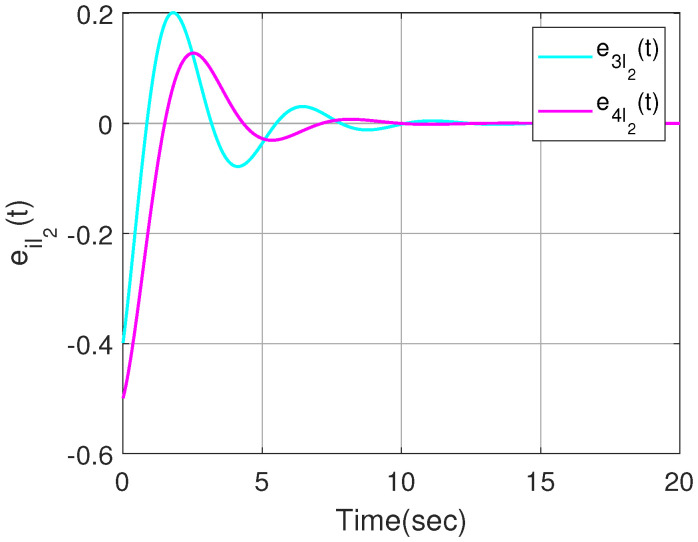
Output trajectories $e_{il_{2}}\left(t\right)$ within the cluster $G_{2}$.

The effectiveness of Theorem 2 can also be demonstrated in the following example. We set ${\hat{x}}_{i}\left(0\right)$ within the interval [−1,1]. By designing an observer-based controller for each heterogeneous agent, Figure 7 and Figure 8 show the state trajectories of errors $\psi \left(t\right)=x_{i}\left(t\right)-{\hat{x}}_{i}\left(t\right)\;\left(i=1,2,3,4\right)$, from which the proposed observer can successfully estimate and utilize the output information of heterogeneous agents. By defining output errors $e_{il_{k}}=y_{i}-y_{l_{k}}\left(i\in G_{k}\right)$ within each cluster, the evolution of the tracking errors of the followers and the leader within each cluster are shown in Figure 9 and Figure 10. The simulation results show that the follower *i* can track the leader $l_{k}\left(i\in G_{k}\right)$.

**Figure 11:**
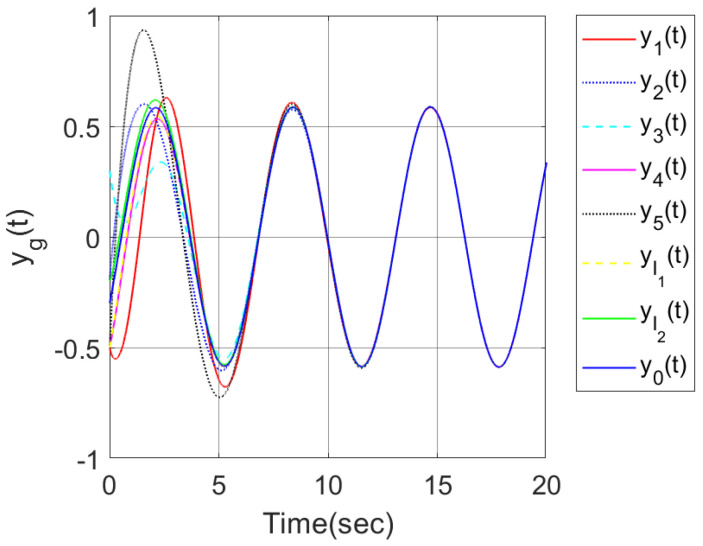
Output trajectories of all agents.

**Figure 12:**
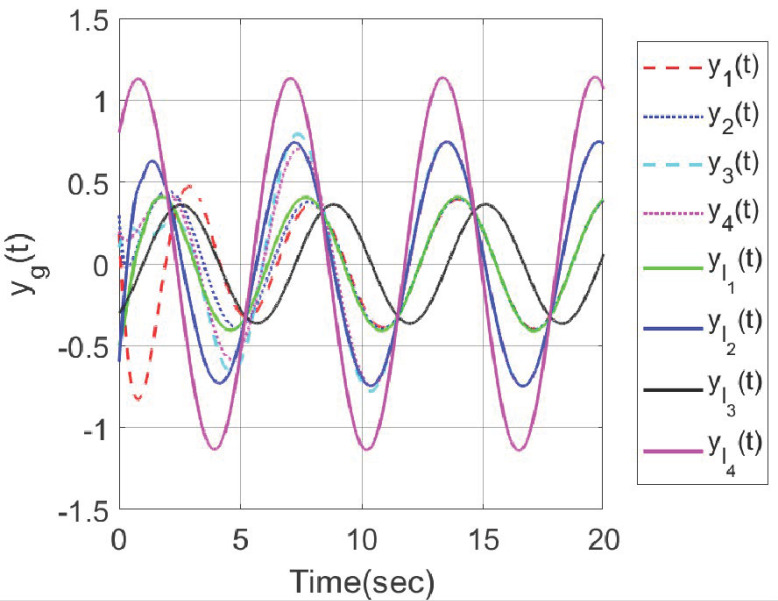
Output trajectories $y_{g}\left(t\right)$ of all agents.

In order to make a comparison, we considered the two control strategies in this paper and the reference [26], where four followers, two internal leaders and one exogenous leader were considered in the clustered network. Under the distributed hybrid control strategy proposed in [26], the output trajectories of all agents were shown in Figure 11, which indicated that all followers can track the output trajectory of the exogenous leader. When an output containment control problem is considered for the multi-agent system in [26], by using the distributed adaptive control strategy proposed in this paper, set $g=1,\cdots ,N,l_{1},\cdots ,l_{M+M_{0}}$, Figure 12 shows that the output trajectories of the four heterogeneous followers enter the desired set spanned by two leaders on output. The abovementioned simulation results demonstrate the effectiveness of the developed hybrid control method.

## 5. Conclusions

In this study, a heterogeneous clustered network framework with multiple leaders was developed with applications in wide-area scenarios and complex tasks. We investigated the intermittent output containment problem of heterogeneous multi-agent systems. Considering that followers may not know the system matrix *S* of the reference leader, we designed an adaptive distributed intermittent controller to estimate the matrix *S* of the leaders. The solution of the regulator equations was obtained using the developed adaptive control algorithm. By introducing the average dwell-time conditions, we applied a common Lyapunov-based function to prove that the developed switched-error system can be exponentially stabilized under aperiodic intermittent control. Linear matrix inequalities were used to compute the controllers, intermittent rates, and other parameters. Simulation examples were presented to verify the effectiveness of the proposed hybrid control strategy. In the future, we will further consider a finite-time containment tracking problem for wide-area networks with cyber-attack and optimal containment control of practical networked systems.

## Data Availability

Not applicable.

## Abbreviations

The following abbreviations are used in this manuscript:

OCC	Output containment control

## Appendix A

**Proof of Theorem 1.** According to Lemmas 1 and 2, there exists a diagonal matrix $\bar{\Xi }>0$ and scalar $\bar{\rho }>0$, such that $\bar{\Xi }\bar{H}+{\bar{H}}^{T}\bar{\Xi }\geq \bar{\rho }\bar{\Xi }$. From (14), we can construct an appropriate Lyapunov function as follows:

(A1) $$\begin{array}{l} V\left(\phi \left(t\right)\right)=\phi^{T}\left(t\right)\left(\bar{\Xi }⊗P\right)\phi \left(t\right), \end{array}$$

where P>0 is the solution of (20). Based on the aperiodic intermittent control, the switched error system (14) can be exponentially stabilized, as shown below. For $t\in [T_{m},T_{m}+\tau_{m}]$, the time derivative of (A1) along the trajectory of system (14) is given as

(A2) $$\begin{array}{lll} \dot{V}\left(\phi \left(t\right)\right) & = & 2\phi^{T}\left(t\right)\left(\bar{\Xi }⊗P\right)\dot{\phi }\left(t\right)\\ & = & \;\phi^{T}\left(t\right)\left(\bar{\Xi }⊗\left(PS\;+\;S^{T}P\right)\;-\;d\left(\bar{\Xi }\bar{H}\;+\;{\bar{H}}^{T}\bar{\Xi }\right)⊗P\right)\phi \left(t\right)\;+\;2\phi^{T}\left(t\right)\left(\bar{\Xi }⊗P\right)(\hat{S}\left(t\right)\phi \left(t\right)\\ & & +\hat{S}\left(t\right)\Phi \left(t\right))\\ & \leq & \phi^{T}\left(t\right)\left(\bar{\Xi }⊗\left(PS+S^{T}P\;-\;d\bar{\rho }P\right)\right)\phi \left(t\right)\;+\;2\phi^{T}\left(t\right)\left(\bar{\Xi }⊗P\right)\left(\hat{S}\left(t\right)\phi \left(t\right)\;+\;\hat{S}\left(t\right)\Phi \left(t\right)\right). \end{array}$$

From (16), this implies that $∥\hat{S}\left(t\right)∥\;\leq \;∥\hat{S}\left(0\right)∥e^{-\gamma_{1}\mu_{0}t}\leq ∥\hat{S}\left(0\right)∥$. By denoting $\nu_{0}=∥\hat{S}\left(0\right)∥>0$, it implies that $2\phi^{T}\left(t\right)\left(\bar{\Xi }⊗P\right)\hat{S}\left(t\right)\phi \left(t\right)\leq 2\nu_{0}\phi^{T}\left(t\right)\left(\bar{\Xi }⊗P\right)\phi \left(t\right)$. From (A2), it can be deduced that $2\phi^{T}\left(t\right)\left(\bar{\Xi }⊗P\right)\hat{S}\left(t\right)\Phi \left(t\right)\leq \theta \phi^{T}\left(t\right)\left(\bar{\Xi }⊗P\right)\phi \left(t\right)+\frac{1}{\theta }∥\bar{\Xi }⊗P∥∥\hat{S}\left(0\right)\Phi \left(0\right){∥}^{2}e^{-(2\gamma_{1}\mu_{0}-2R\left(\sigma_{max}\left(S\right)\right))t}$ with θ>0 and $2\gamma_{1}\mu_{0}-2R\left(\sigma_{max}\left(S\right)\right)>0$. Moreover, letting $\mu =\frac{1}{\theta }∥\bar{\Xi }⊗P∥∥\hat{S}{\left(0\right)\Phi \left(0\right)∥}^{2}>0$ and $\sigma =2\gamma_{1}\mu_{0}-2R\left(\sigma_{max}\left(S\right)\right)>0$, we have

(A3) $$\begin{array}{ll} \dot{V}\left(\phi \left(t\right)\right) & \leq \;-\;\phi^{T}\left(t\right)\left(\bar{\Xi }⊗Q\right)\phi \left(t\right)\;+\;\phi^{T}\left(t\right)\left(\bar{\Xi }⊗\left(PS\;+\;S^{T}P\;+\;\theta P\;+\;2\nu_{0}P\;+\;Q\;-\;d\bar{\rho }P\right)\right)\phi \left(t\right)\;+\;\mu e^{-\sigma t}\\ \; & \leq -\phi^{T}\left(t\right)\left(\bar{\Xi }⊗Q\right)\phi \left(t\right)+\mu e^{-\sigma t}. \end{array}$$

From (A3), by denoting $\alpha =\frac{\lambda_{min}\left(Q\right)}{\lambda_{max}\left(P\right)}$, it implies that $\dot{V}\left(\phi \left(t\right)\right)<-\alpha \phi^{T}\left(t\right)\left(\bar{\Xi }⊗P\right)\phi \left(t\right)+\mu e^{-\sigma t}$, from which we can deduce that $V\left(\phi \left(t\right)\right)\leq V\left(\phi \left(T_{0}\right)\right)e^{-\alpha t}+\mu \left(T_{0}\right)\int_{0}^{t}e^{-\alpha (t-\tau )}e^{-\sigma \tau }d\tau$. Letting α>σ, we obtain

(A4) $$\begin{array}{l} V\left(\phi \left(t\right)\right)\leq \left(V\left(\phi \left(T_{m}\right)\right)-\frac{\mu (T_{m})}{\alpha -\sigma }\right)e^{-\alpha (t-T_{m})}+\frac{\mu (T_{m})}{\alpha -\sigma }e^{-\sigma (t-T_{m})}.\;t\in \left[T_{m},T_{m}+\tau_{m}\right] \end{array}$$

Similarly, for $t\in (T_{m}+\tau_{m},T_{m+1})$, it follows from (A1)–(A4) that

(A5) $$\begin{array}{lll} \dot{V}\left(\phi \left(t\right)\right) & = & 2\phi^{T}\left(t\right)\left(\bar{\Xi }⊗P\right)\dot{\phi }\left(t\right)\\ & = & \;\phi^{T}\left(t\right)\left(\bar{\Xi }⊗\beta P\right)\phi \left(t\right)\;+\;\phi^{T}\left(t\right)\left(\bar{\Xi }⊗\left(PS\;+\;S^{T}P\;-\;\beta P\right)\right)\phi \left(t\right)\;+\;2\phi^{T}\left(t\right)\left(\bar{\Xi }⊗P\right)(\hat{S}\left(t\right)\phi \left(t\right)\\ & & +\hat{S}\left(t\right)\Phi \left(t\right))\\ & \leq & \beta \phi^{T}\left(t\right)\left(\bar{\Xi }⊗P\right)\phi \left(t\right)+\mu e^{-\sigma t}, \end{array}$$

where β>0. From (A5), this implies that $V\left(\phi \left(t\right)\right)\leq V\left(\phi \left(T_{0}\right)\right)e^{\beta t}+\mu \left(T_{0}\right)\int_{0}^{t}e^{\beta (t-\tau )}e^{-\sigma \tau }d\tau$, that is,

(A6) $$\begin{array}{l} V\left(\phi \left(t\right)\right)\leq \left(V\left(\phi \left(T_{0}\right)\right)+\frac{\mu (T_{0})}{\sigma +\beta }\right)e^{\beta t}-\frac{\mu (T_{0})}{\sigma +\beta }e^{-\sigma t}. \end{array}$$

Furthermore, it holds that

(A7) $$\begin{array}{ll} V(\phi (t))\leq & \left(V\left(\phi \left(T_{m}+\tau_{m}\right)\right)+\frac{\mu (T_{m}+\tau_{m})}{\sigma +\beta }\right)e^{\beta (t-T_{m}-\tau_{m})}\\ & -\frac{\mu (T_{m}+\tau_{m})}{\sigma +\beta }e^{-\sigma (t-T_{m}-\tau_{m})}.\;t\in \left(T_{m}+\tau_{m},T_{m+1}\right) \end{array}$$

However, using the developed aperiodic intermittent control approach, the stability of the switched error system (14) must be proven exponentially. From (A4) and (A7), a form of exponential inequalities is obtained for $t\in [T_{m},T_{m+1})$. Thus, the following derivation process is implemented according to intermittent intervals. Letting $\varepsilon =\frac{\mu }{\alpha -\sigma }=\frac{\mu }{\sigma +\beta }$, it follows from (A4) that

(A8) $$\begin{array}{ll} V(\phi (t)) & \leq \left(V\left(\phi \left(T_{0}\right)\right)-\varepsilon \left(T_{0}\right)\right)e^{-\alpha (t-T_{0})}+\varepsilon \left(T_{0}\right)e^{-\sigma (t-T_{0})}\\ \; & \leq V\left(\phi \left(T_{0}\right)\right)e^{-\alpha (t-T_{0})}+\varepsilon \left(T_{0}\right)e^{-\sigma (t-T_{0})}\\ \; & \leq V\left(\phi \left(T_{0}\right)\right)+\varepsilon \left(T_{0}\right).\;t\in \left[T_{0},T_{0}+\tau_{0}\right] \end{array}$$

From (A4) and (A7), we obtain

(A9) $$\begin{array}{ll} V(\phi (t)) & \leq \left(V\left(\phi \left(T_{0}+\tau_{0}\right)\right)+\varepsilon \left(T_{0}+\tau_{0}\right)\right)e^{\beta (t-T_{0}-\tau_{0})}-\varepsilon \left(T_{0}+\tau_{0}\right)e^{-\sigma (t-T_{0}-\tau_{0})}\\ & \leq \left(V\left(\phi \left(T_{0}\right)\right)e^{-\alpha \tau_{0}}+\varepsilon \left(T_{0}\right)e^{-\sigma \tau_{0}}\right)e^{\beta (t-T_{0}-\tau_{0})}+\varepsilon \left(T_{0}\right)e^{-\sigma \tau_{0}+\beta \left(t-T_{0}-\tau_{0}\right)}\\ & \leq V\left(\phi \left(T_{0}\right)\right)e^{-\left(\alpha +\beta \right)\tau_{0}+\beta \left(T_{1}-T_{0}\right)}+2\varepsilon \left(T_{0}\right)e^{-\left(\sigma +\beta \right)\tau_{0}+\beta \left(T_{1}-T_{0}\right)}.\;t\in \left(T_{0}+\tau_{0},T_{1}\right) \end{array}$$

Similar to (A9), we have

(A10) $$\begin{array}{lll} V(\phi (t)) & \leq & \left(V\left(\phi \left(T_{1}\right)\right)-\varepsilon \left(T_{1}\right)\right)e^{-\alpha (t-T_{1})}+\varepsilon \left(T_{1}\right)e^{-\sigma (t-T_{1})}\\ & \leq & V\left(\phi \left(T_{0}\right)\right)e^{-\left(\alpha +\beta \right)\tau_{0}-\alpha t+\beta \left(T_{1}-T_{0}\right)}+2\varepsilon \left(T_{0}\right)e^{-\left(\sigma +\beta \right)\tau_{0}-\alpha t+\beta \left(T_{1}-T_{0}\right)}\\ \; & \; & +\varepsilon \left(T_{0}\right)e^{-\sigma (t-T_{0})}\\ \; & \leq & V\left(\phi \left(T_{0}\right)\right)e^{-\left(\alpha +\beta \right)\tau_{0}+\beta \left(T_{1}-T_{0}\right)}+2\varepsilon \left(T_{0}\right)e^{-\left(\sigma +\beta \right)\tau_{0}+\beta \left(T_{1}-T_{0}\right)}\\ \; & \; & +\varepsilon \left(T_{0}\right)e^{-\sigma (T_{1}-T_{0})}.\;t\in \left[T_{1},T_{1}+\tau_{1}\right] \end{array}$$

Similarly, from (A9) and (A10), we obtain

(A11) $$\begin{array}{lll} V(\phi (t)) & \leq & V\left(\phi \left(T_{0}\right)\right)e^{-\left(\alpha +\beta \right)\left(\tau_{0}+\tau_{1}\right)+\beta \left(t-T_{0}\right)}+2\varepsilon \left(T_{0}\right)e^{-\left(\sigma +\beta \right)\tau_{0}-\left(\alpha +\beta \right)\tau_{1}+\beta \left(T_{2}-T_{0}\right)}\\ \; & \; & +2\varepsilon \left(T_{0}\right)e^{-\sigma \left(T_{1}+\tau_{1}-T_{0}\right)+\beta \left(t-T_{1}-\tau_{1}\right)}\\ \; & \leq & V\left(\phi \left(T_{0}\right)\right)e^{-\left(\alpha +\beta \right)\left(\tau_{0}+\tau_{1}\right)+\beta \left(T_{2}-T_{0}\right)}+2\varepsilon \left(T_{0}\right)e^{-\left(\sigma +\beta \right)\tau_{0}-\left(\alpha +\beta \right)\tau_{1}+\beta \left(T_{2}-T_{0}\right)}\\ \; & \; & +2\varepsilon \left(T_{0}\right)e^{-\sigma \left(T_{1}+\tau_{1}-T_{0}\right)+\beta \left(T_{2}-T_{1}-\tau_{1}\right)}.\;t\in \left(T_{1}+\tau_{1},T_{2}\right) \end{array}$$

From (A10) and (A11), we define $V\left(\phi \left(t\right)\right)=\hat{V}\left(\phi \left(t\right)\right)+\Psi \left(t\right)$. Then, according to intermittent intervals, the following derivation processes are derived for $\hat{V}\left(\phi \left(t\right)\right)$ and Ψ(t). $\hat{V}\left(\phi \left(t\right)\right)$, is analyzed as follows. When $t\in [T_{m},T_{m}+\tau_{m}]$, it implies that

(A12) $$\begin{array}{ll} \hat{V}\left(\phi \left(t\right)\right) & \leq \hat{V}\left(\phi \left(T_{m}\right)\right)e^{-\alpha (t-T_{m})}\\ & \leq \hat{V}\left(\phi \left(T_{0}\right)\right)e^{-\alpha \sum_{s=0}^{m-1}\tau_{s}-\alpha \left(t-T_{m}\right)+\beta \sum_{s=1}^{m}\left(T_{s}-T_{s-1}-\tau_{s-1}\right)}\\ & \leq \hat{V}\left(\phi \left(T_{0}\right)\right)e^{-\alpha \sum_{s=0}^{m-1}\tau_{s}+\beta \sum_{s=1}^{m}\left(T_{s}-T_{s-1}-\tau_{s-1}\right)}.m\geq 1 \end{array}$$

Similarly, when $t\in (T_{m}+\tau_{m},T_{m+1})$, we obtain

(A13) $$\begin{array}{ll} \hat{V}\left(\phi \left(t\right)\right) & \leq \hat{V}\left(\phi \left(T_{m}+\tau_{m}\right)\right)e^{\beta (t-T_{m}-\tau_{m})}\\ & \leq \hat{V}\left(\phi \left(T_{0}\right)\right)e^{-\alpha \sum_{s=0}^{m}\tau_{s}+\beta \sum_{s=1}^{m}\left(T_{s}-T_{s-1}-\tau_{s-1}\right)+\beta \left(t-T_{m}-\tau_{m}\right)}\\ & \leq \hat{V}\left(\phi \left(T_{0}\right)\right)e^{-\alpha \sum_{s=0}^{m}\tau_{s}+\beta \sum_{s=1}^{m+1}\left(T_{s}-T_{s-1}-\tau_{s-1}\right)}.m\geq 1 \end{array}$$

Under Assumption 5, for $m\geq m^{*}$, we can conclude that $-\alpha \sum_{s=0}^{m-1}\tau_{s}+\beta \sum_{s=1}^{m}\left(T_{s}-T_{s-1}-\tau_{s-1}\right)<0$ if $\alpha \varrho_{1}>\beta \left(\varrho_{2}-\varrho_{1}\right)$. Moreover, $\varrho_{1}m\leq \sum_{s=0}^{m-1}\tau_{s}$ and $\frac{T_{m}-T_{0}}{\varrho_{2}}\leq m$ can be derived. Thus, using (A12), we obtain

(A14) $$\begin{array}{ll} \hat{V}\left(\phi \left(t\right)\right) & \leq \hat{V}\left(\phi \left(T_{0}\right)\right)e^{-\alpha \sum_{s=0}^{m-1}\tau_{s}+\beta \sum_{s=1}^{m}\left(T_{s}-T_{s-1}-\tau_{s-1}\right)}\\ & \leq \hat{V}\left(\phi \left(T_{0}\right)\right)e^{-\left(\alpha +\beta \right)\sum_{s=0}^{m-1}\tau_{s}+\beta \left(T_{m}-T_{0}\right)}\\ & \leq \hat{V}\left(\phi \left(T_{0}\right)\right)e^{-\frac{\left(\alpha +\beta \right)\varrho_{1}\left(T_{m}-T_{0}\right)}{\varrho_{2}}+\beta \left(T_{m}-T_{0}\right)}.\;m\geq m^{*} \end{array}$$

Similarly, from (A13), we obtain

(A15) $$\begin{array}{ll} \hat{V}\left(\phi \left(t\right)\right) & \leq \hat{V}\left(\phi \left(T_{0}\right)\right)e^{-\alpha \sum_{s=0}^{m}\tau_{s}+\beta \sum_{s=1}^{m+1}\left(T_{s}-T_{s-1}-\tau_{s-1}\right)}\\ & \leq \hat{V}\left(\phi \left(T_{0}\right)\right)e^{-\left(\alpha +\beta \right)\sum_{s=0}^{m}\tau_{s}+\beta \left(T_{m+1}-T_{0}\right)}\\ & \leq \hat{V}\left(\phi \left(T_{0}\right)\right)e^{-\frac{\left(\alpha +\beta \right)\varrho_{1}\left(T_{m+1}-T_{0}\right)}{\varrho_{2}}+\beta \left(T_{m+1}-T_{0}\right)}.\;m\geq m^{*} \end{array}$$

Next, similar derivations are obtained for Ψ(t). Because $\varepsilon (T_{0})>0$, σ>0, and α>σ, it can be deduced that $\varepsilon \left(T_{0}\right)e^{-\sigma (T_{1}-T_{0})}\leq 2\varepsilon \left(T_{0}\right)e^{-\sigma (T_{1}-T_{0})}$ and $2\varepsilon \left(T_{0}\right)e^{-\left(\sigma +\beta \right)\tau_{0}-\left(\alpha +\beta \right)\tau_{1}}\leq 2\varepsilon \left(T_{0}\right)e^{-\left(\sigma +\beta \right)\left(\tau_{0}+\tau_{1}\right)}$. Thus, from (A10), it implies that

(A16) $$\begin{array}{ll} \Psi (t)\leq & 2\varepsilon \left(T_{0}\right)e^{-\sigma \sum_{s=0}^{m-1}\tau_{s}+\beta \sum_{s=1}^{m}\left(T_{s}-T_{s-1}-\tau_{s-1}\right)}\\ & \left(1+e^{-\left(\sigma +\beta \right)\left(T_{1}-T_{0}-\tau_{0}\right)}+\cdots +e^{-\left(\sigma +\beta \right)\sum_{s=1}^{m}\left(T_{s}-T_{s}-\tau_{s}\right)}\right).\;t\in \left[T_{m},T_{m}+\tau_{m}\right] \end{array}$$

Similarly, from (A11), we obtain

(A17) $$\begin{array}{ll} \Psi (t)\leq & 2\varepsilon \left(T_{0}\right)e^{-\sigma \sum_{s=0}^{m}\tau_{s}+\beta \sum_{s=1}^{m+1}\left(T_{s}-T_{s-1}-\tau_{s-1}\right)}\\ & \left(1+e^{-\left(\sigma +\beta \right)\left(T_{1}-T_{0}-\tau_{0}\right)}+\cdots +e^{-\left(\sigma +\beta \right)\sum_{s=1}^{m}\left(T_{s}-T_{s}-\tau_{s}\right)}\right).\;t\in \left(T_{m}+\tau_{m},T_{m+1}\right) \end{array}$$

The condition $\sigma \varrho_{1}>\beta \left(\varrho_{2}-\varrho_{1}\right)$ implies that $-\sigma \sum_{s=0}^{m-1}\tau_{s}+\beta \sum_{s=1}^{m}\left(T_{s}-T_{s-1}-\tau_{s-1}\right)<0$. Under Assumption 5, using (A16) and (A17), we deduce

(A18) $$\begin{array}{ll} \Psi (t)\leq & 2\varepsilon \left(T_{0}\right)e^{-\sigma \sum_{s=0}^{m-1}\tau_{s}+\beta \sum_{s=1}^{m}\left(T_{s}-T_{s-1}-\tau_{s-1}\right)}\\ & \left(1+e^{-\left(\sigma +\beta \right)\left(T_{1}-T_{0}-\tau_{0}\right)}+\cdots +e^{-\left(\sigma +\beta \right)\sum_{s=1}^{m}\left(T_{s}-T_{s}-\tau_{s}\right)}\right)\\ \leq & 2\varepsilon \left(T_{0}\right)e^{-\sigma \sum_{s=0}^{m-1}\tau_{s}+\beta \sum_{s=1}^{m}\left(T_{s}-T_{s-1}-\tau_{s-1}\right)}\\ & \left(1+e^{-(\sigma +\beta )h}+\cdots +e^{-\left(\sigma +\beta \right)\sum_{s=1}^{m}h}\right)\\ \leq & 2\varepsilon \left(T_{0}\right)e^{-\frac{\left(\sigma +\beta \right)\varrho_{1}\left(T_{m}-T_{0}\right)}{\varrho_{2}}+\beta \left(T_{m}-T_{0}\right)}\left(\frac{1-e^{-mh(\sigma +\beta )}}{1-e^{-(\sigma +\beta )}}\right). \end{array}$$

Combining (A14), (A15), and (A18), it follows from (A10) and (A11) that for $m\geq m^{*}$

(A19) $$\begin{array}{ll} V(\phi (t))\leq & V\left(\phi \left(T_{0}\right)\right)e^{\left(-\frac{\left(\alpha +\beta \right)\varrho_{1}}{\varrho_{2}}+\beta \right)\left(T_{m}-T_{0}\right)}\\ & +2\varepsilon \left(T_{0}\right)e^{\left(-\frac{\left(\sigma +\beta \right)\varrho_{1}}{\varrho_{2}}+\beta \right)\left(T_{m}-T_{0}\right)}\left(\frac{1-e^{-mh(\sigma +\beta )}}{1-e^{-(\sigma +\beta )}}\right),\;t\in \left[T_{m},T_{m+1}\right) \end{array}$$

where $-\frac{\left(\alpha +\beta \right)\varrho_{1}}{\varrho_{2}}+\beta <0$ and $-\frac{\left(\sigma +\beta \right)\varrho_{1}}{\varrho_{2}}+\beta <0$ are inferred according to Assumption 5. Let $T_{0}=0$, $e^{\left(-\frac{\left(\alpha +\beta \right)\varrho_{1}}{\varrho_{2}}+\beta \right)T_{m}}\to 0$ and $e^{\left(-\frac{\left(\sigma +\beta \right)\varrho_{1}}{\varrho_{2}}+\beta \right)T_{m}}\to 0$ are obtained as $T_{m}\to \infty$. Meanwhile, as $T_{m}\to \infty$, mh approaches *∞*, which implies that $e^{-mh(\sigma +\beta )}\to 0$. From (A19), $V\left(\phi \left(t\right)\right)\leq V\left(\phi \left(0\right)\right)e^{(-\frac{\left(\alpha +\beta \right)\varrho_{1}}{\varrho_{2}}+\beta )t}+\frac{2\varepsilon (0)}{1-e^{-(\sigma +\beta )}}e^{(-\frac{\left(\sigma +\beta \right)\varrho_{1}}{\varrho_{2}}+\beta )t}$ with $m>m^{*}$, which implies that $\lim_{t\to \infty }V\left(\phi \left(t\right)\right)=0$ and $\lim_{t\to \infty }\phi \left(t\right)=0$. Then, we say that the developed switched error system (14) is stabilized, which means that the proposed distributed aperiodic intermittent controller (6) can achieve containment tracking. Considering ${\bar{G}}_{L}$, we denote the local output error for the $l_{k}$th leader as

(A20) $$\begin{array}{l} {\hat{e}}_{l_{k}}\left(t\right)=\sum_{v\in L_{M}}a_{l_{k}l_{v}}\left(y_{l_{v}}\left(t\right)-y_{l_{k}}\left(t\right)\right)+\sum_{r\in L_{M_{0}}}\omega_{l_{k}l_{r}^{0}}\left(y_{l_{r}^{0}}\left(t\right)-y_{l_{k}}\left(t\right)\right). \end{array}$$

We define $e_{y}\left(t\right)={\left[{\hat{e}}_{l_{1}}^{T}\left(t\right),{\hat{e}}_{l_{2}}^{T}\left(t\right),\cdots ,{\hat{e}}_{l_{M}}^{T}\left(t\right)\right]}^{T}\in R^{Mp}$, $y\left(t\right)={\left[y_{l_{1}}^{T}\left(t\right),y_{l_{2}}^{T}\left(t\right),\cdots ,y_{l_{M}}^{T}\left(t\right)\right]}^{T}$$\in R^{Mp}$, and ${\bar{y}}_{r}\left(t\right)=1_{M}⊗y_{l_{r}^{0}}\left(t\right)$. Subsequently, we can rewrite the error $e_{y}\left(t\right)$ as $e_{y}\left(t\right)=-\sum_{r\in L_{M_{0}}}\left(H_{r}⊗I_{p}\right)\left(y\left(t\right)-{\bar{y}}_{r}\left(t\right)\right)$, which can be translated as follows:

(A21) $$\begin{array}{l} \hat{e}\left(t\right)=y\left(t\right)-{\left(\sum_{\bar{r}\in L_{M_{0}}}\left(H_{\bar{r}}⊗I_{p}\right)\right)}^{-1}\sum_{r\in L_{M_{0}}}\left(H_{r}⊗I_{p}\right){\bar{y}}_{r}\left(t\right), \end{array}$$

where $e_{y}=-\sum_{r\in L_{M_{0}}}\hat{e}\left(t\right)$. From (A21), as t→∞ and error $\hat{e}\left(t\right)$ approaches zero, we obtain

(A22) $$\begin{array}{ll} y(t) & ={\left(\sum_{\bar{r}\in L_{M_{0}}}\left(H_{\bar{r}}⊗I_{p}\right)\right)}^{-1}\sum_{r\in L_{M_{0}}}\left(H_{r}⊗I_{p}\right){\bar{y}}_{r}\left(t\right)\\ & =\sum_{r\in L_{M_{0}}}(({\left(\sum_{\bar{r}\in L_{M_{0}}}H_{\bar{r}}\right)}^{-1}⊗I_{p})\left(H_{r}⊗I_{p}\right)(1_{M}⊗y_{l_{r}^{0}}\left(t\right)))\\ & =\sum_{r\in L_{M_{0}}}(({\left(\sum_{\bar{r}\in L_{M_{0}}}H_{\bar{r}}\right)}^{-1}H_{r}1_{M})⊗y_{l_{r}^{0}}\left(t\right)), \end{array}$$

where $H_{r}$ and ${\left(\sum_{r\in L_{M_{0}}}H_{r}\right)}^{-1}$ are non-negative according to Lemma 1. This implies that $\left({\left(\sum_{\bar{r}\in L_{M_{0}}}H_{\bar{r}}\right)}^{-1}H_{r}1_{M}\right)\geq 0$. Moreover, because

(A23) $$\begin{array}{ll} \sum_{r\in L_{M_{0}}}\left({\left(\sum_{\bar{r}\in L_{M_{0}}}H_{\bar{r}}\right)}^{-1}H_{r}1_{M}\right) & ={\left(\sum_{\bar{r}\in L_{M_{0}}}H_{\bar{r}}\right)}^{-1}\left(\sum_{r\in L_{M_{0}}}H_{r}1_{M}\right)\\ & ={\left(\sum_{\bar{r}\in L_{M_{0}}}H_{\bar{r}}\right)}^{-1}\left(\sum_{r\in L_{M_{0}}}H_{r}\right)1_{M}\\ & =1_{M}, \end{array}$$

the sum of each row of $\sum_{r\in L_{M_{0}}}\left({\left(\sum_{\bar{r}\in L_{M_{0}}}H_{\bar{r}}\right)}^{-1}H_{r}1_{M}\right)$ is 1. Furthermore, because $\lim_{t\to \infty }\hat{e}\left(t\right)=D\lim_{t\to \infty }\phi \left(t\right)=0$, we conclude that $\lim_{t\to \infty }\left(y\left(t\right)-\sum_{r\in L_{M_{0}}}\left({\left(\sum_{\bar{r}\in L_{M_{0}}}H_{\bar{r}}\right)}^{-1}H_{r}1_{M}\right)⊗y_{l_{r}^{0}}\left(t\right)\right)=0$. Furthermore, we define $\alpha_{r}={\left[\alpha_{1r}^{T},\alpha_{2r}^{T},\cdots ,\alpha_{Mr}^{T}\right]}^{T}$. Thus, $\sum_{r\in L_{M_{0}}}\left({\left(\sum_{\bar{r}\in L_{M_{0}}}H_{\bar{r}}\right)}^{-1}H_{r}1_{M}\right)=\sum_{r\in L_{M_{0}}}\alpha_{r}⊗y_{l_{r}^{0}}\left(t\right)$, whose rows are denoted as $y_{l_{k}}\left(t\right)-\sum_{r\in L_{M_{0}}}\alpha_{kr}y_{l_{r}^{0}}\left(t\right)$. From (A23), this implies that $\sum_{r\in L_{M_{0}}}\alpha_{r}=1_{M}$, under which $\lim_{t\to \infty }\left(y_{l_{k}}\left(t\right)-\sum_{r\in L_{M_{0}}}\alpha_{kr}y_{l_{r}^{0}}\left(t\right)\right)=0$. According to Definitions 1 and 2, *M* internal leaders can be guided into the convex set formed by $M_{0}$ leaders on output; that is, output containment is achieved for the leaders. □

## Appendix B

**Proof of Theorem 2.** First, we must prove that $e_{k}\left(t\right)$ is exponentially stable. From (3), we obtain $∥x_{k}\left(t\right)∥\;\leq \;∥x_{k}\left(0\right)∥e^{R(\sigma_{max}\left(S\right))t}$. Using Lemma 3, it follows from (3) and (15) that

(A24) $$\begin{array}{l} ∥S_{k}\left(t\right)x_{k}\left(t\right)∥\;\leq \;∥S_{k}\left(0\right)-\frac{\Lambda_{k}}{\gamma_{2}\mu_{k}\;-\;\gamma_{1}\mu_{0}}∥e^{(-\gamma_{2}\mu_{k}\;+\;R\left(\sigma_{max}\left(S\right)\right))t}\;+\;\frac{∥\Lambda_{k}∥}{\gamma_{2}\mu_{k}-\gamma_{1}\mu_{0}}e^{(-\gamma_{1}\mu_{0}+R\left(\sigma_{max}\left(S\right)\right))t}, \end{array}$$

which implies that $\lim_{t\to \infty }S_{k}\left(t\right)x_{k}\left(t\right)=0$ considering (17). Following Theorem 1, $\lim_{t\to \infty }\hat{S}e_{L}\left(t\right)=0$ is obtained, which implies that $\lim_{t\to \infty }{\bar{H}}_{k}e_{L}\left(t\right)=0$. Furthermore, using Lemma 3, it implies that ${\hat{S}}_{k}\left(t\right)$ is bounded and $\lim_{t\to \infty }S_{k}\left(t\right)=0$, which further implies that $\lim_{t\to \infty }\left(S_{k}\left(t\right)-{\hat{S}}_{k}\left(t\right)\right)<0$. Thus, we confirm that (15) is stable, which means that the proposed distributed hybrid controller can achieve consensus tracking within each cluster. Next, output consensus tracking within each cluster $G_{k}$ must be realized based on the intermittent control and adaptive algorithm. By introducing the adaptive algorithm, it follows from (8) that

(A25) $$u_{i}\left(t\right)=K_{1i}{\hat{x}}_{i}\left(t\right)+K_{2i}\left(t\right)z_{i}\left(t\right),\;i\in G_{k}$$

where $K_{2i}\left(t\right)=U_{i}\left(t\right)-K_{1i}\Pi_{i}\left(t\right)$. From (7) and (8), by denoting $\psi_{i}\left(t\right)=x_{i}\left(t\right)-{\hat{x}}_{i}\left(t\right)$, we obtain $\dot{\psi }\left(t\right)=A_{i}+F_{i}C_{i}$, implying that $\lim_{t\to \infty }\psi \left(t\right)=0$ by choosing appropriate $F_{i}$, which satisfies that $A_{i}+F_{i}C_{i}$ is a Hurwitz matrix. Furthermore, we define ${\bar{x}}_{i}\left(t\right)=x_{i}\left(t\right)-\Pi_{i}^{*}x_{l_{k}}\left(t\right)$. By applying the intermittent control mechanism, the dynamics of φ(t) can be derived according to the general aperiodic intermittent intervals. For $t\in [T_{m},T_{m}+\tau_{m}]$, using (3), (7), and (8), we obtain

(A26) $$\begin{array}{ccc} {\dot{\bar{x}}}_{i}\left(t\right) & = & {\dot{x}}_{i}\left(t\right)-\Pi_{i}^{*}{\dot{x}}_{l_{k}}\left(t\right)\\ & = & A_{i}x_{i}\left(t\right)+B_{i}\left(K_{1i}x_{i}\left(t\right)+K_{2i}\left(t\right)z_{i}\left(t\right)\right)+B_{i}K_{1i}\left({\hat{x}}_{i}\left(t\right)-x_{i}\left(t\right)\right)-\Pi_{i}^{*}Sx_{l_{k}}\left(t\right)\\ & & -\Pi_{i}^{*}\left(S_{l_{k}}\left(t\right)-S\right)x_{l_{k}}\left(t\right)+\Pi_{i}d\left({\bar{H}}_{k}⊗I_{n_{0}}\right)\phi \left(t\right). \end{array}$$

From (4), letting $K_{2i}=U_{i}^{*}-K_{1i}\Pi_{i}^{*}$, we obtain

(A27) $$\begin{array}{ccc} {\dot{\bar{x}}}_{l_{k}}\left(t\right) & = & \left(A_{i}+B_{i}K_{1i}\right)x_{i}\left(t\right)+\left(B_{i}U_{i}\left(t\right)-B_{i}K_{1i}\Pi_{i}\left(t\right)\right)z_{i}\left(t\right)-\left(A_{i}\Pi_{i}^{*}+B_{i}U_{i}^{*}\right)x_{l_{k}}\left(t\right)\\ & & -\Pi_{i}^{*}\left(S_{l_{k}}\left(t\right)-S\right)x_{l_{k}}\left(t\right)+B_{i}K_{1i}\left({\hat{x}}_{i}\left(t\right)-x_{i}\left(t\right)\right)+\Pi_{i}^{*}d\left({\bar{H}}_{k}⊗I_{n_{0}}\right)\phi \left(t\right)\\ & = & \left(A_{i}+B_{i}K_{1i}\right){\bar{x}}_{i}\left(t\right)+B_{i}K_{1i}\left(\Pi_{i}^{*}-\Pi_{i}\left(t\right)\right)z_{i}\left(t\right)+B_{i}\left(U_{i}\left(t\right)-U_{i}^{*}\right)z_{i}\left(t\right)\\ & & +B_{i}K_{2i}^{*}\left(z_{i}\left(t\right)-x_{l_{k}}\left(t\right)\right)-\Pi_{i}^{*}\left(S_{l_{k}}\left(t\right)-S\right)x_{l_{k}}\left(t\right)+B_{i}K_{1i}\left({\hat{x}}_{i}\left(t\right)-x_{i}\left(t\right)\right)\\ & & +\Pi_{i}^{*}d\left({\bar{H}}_{k}⊗I_{n_{0}}\right)\phi \left(t\right). \end{array}$$

Similarly, when $t\in (t_{c}+\tau_{c},t_{c+1})$, we obtain

(A28) $$\begin{array}{ll} {\dot{\bar{x}}}_{i}\left(t\right)= & \left(A_{i}+B_{i}K_{1i}\right){\bar{x}}_{i}\left(t\right)+B_{i}K_{1i}\left(\Pi_{i}^{*}-\Pi_{i}\left(t\right)\right)z_{i}\left(t\right)+B_{i}\left(U_{i}\left(t\right)-U_{i}^{*}\right)z_{i}\left(t\right)\\ & +B_{i}K_{2i}^{*}\left(z_{i}\left(t\right)-x_{l_{k}}\left(t\right)\right)-\Pi_{i}^{*}\left(S_{l_{k}}\left(t\right)-S\right)x_{l_{k}}\left(t\right)+B_{i}K_{1i}\left({\hat{x}}_{i}\left(t\right)-x_{i}\left(t\right)\right). \end{array}$$

From (A19) and (A24), it implies that $\lim_{t\to \infty }\phi \left(t\right)=0$ and $\lim_{t\to \infty }e_{k}\left(t\right)=0$, which further implies that $\lim_{t\to \infty }\left(z_{i}\left(t\right)-x_{l_{k}}\left(t\right)\right)=0$. Using Lemma 3, we obtain $\lim_{t\to \infty }\left(S_{l_{k}}\left(t\right)-S\right)=0$. Then, $\lim_{t\to \infty }\left(U_{i}\left(t\right)-U_{i}^{*}\right)=0$ and $\lim_{t\to \infty }\left(\Pi_{i}^{*}-\Pi_{i}\left(t\right)\right)=0$ are obtained using Lemma 4. From (A26)–(A28), we conclude that $\lim_{t\to \infty }{\bar{x}}_{i}\left(t\right)=0$ if $A_{i}+B_{i}K_{1i}$ is a Hurwitz matrix. Thus, as t→∞, the output error is denoted as follows:

(A29) $$\lim_{t\to \infty }\left(y_{i}\left(t\right)-y_{l_{k}}\left(t\right)\right)=\lim_{t\to \infty }\left(C_{i}{\bar{x}}_{i}\left(t\right)\right)=0.$$

Thus, output consensus is achieved within each cluster $G_{k}$. From (A19) and (A29), we obtain $\lim_{t\to \infty }dist\left(y_{l_{k}}\left(t\right)-Co\left(Y\left(t\right)\right)\right)=0$ and $\lim_{t\to \infty }\left(y_{i}\left(t\right)-y_{l_{k}}\left(t\right)\right)=0$. Furthermore, following Definition 2, we infer that

(A30) $$\lim_{t\to \infty }\left(y_{\ell }\left(t\right)-Co\left(Y\left(t\right)\right)\right)=0\;\left(\ell =1,\cdots ,N,l_{1},\cdots ,l_{M}\right).$$

Consequently, leader-following output containment is achieved for the developed clustered network. □
